# Tandem Mass Tag labelling quantitative acetylome analysis of differentially modified proteins during mycoparasitism of *Clonostachys chloroleuca* 67–1

**DOI:** 10.1038/s41598-021-01956-2

**Published:** 2021-11-17

**Authors:** Na Jiang, Binna Lv, Haixia Wu, Shidong Li, Manhong Sun

**Affiliations:** Institute of Plant Protection, Chinese Academy of Agricultural Sciences, Beijing, 100193 China

**Keywords:** Biochemistry, Biological techniques, Biotechnology, Genetics, Microbiology, Molecular biology

## Abstract

Lysine acetylation (Kac) is an important post-translational modification (PTM) of proteins in all organisms, but its functions have not been extensively explored in filamentous fungi. In this study, a Tandem Mass Tag (TMT) labelling lysine acetylome was constructed, and differentially modified Kac proteins were quantified during mycoparasitism and vegetative growth in the biocontrol fungus *Clonostachys chloroleuca* 67–1, using liquid chromatography-tandem mass spectrometry (LC–MS/MS). A total of 1448 Kac sites were detected on 740 Kac proteins, among which 126 sites on 103 proteins were differentially regulated. Systematic bioinformatics analyses indicate that the modified Kac proteins were from multiple subcellular localizations and involved in diverse functions including chromatin assembly, glycometabolism and redox activities. All Kac sites were characterized by 10 motifs, including the novel CxxKac motif. The results suggest that Kac proteins may have effects of broadly regulating protein interaction networks during *C. chloroleuca* parasitism to *Sclerotinia sclerotiorum* sclerotia. This is the first report of a correlation between Kac events and the biocontrol activity of *C. chloroleuca*. Our findings provide insight into the molecular mechanisms underlying *C. chloroleuca* control of plant fungal pathogens regulated by Kac proteins.

## Introduction

Lysine acetylation (Kac) is a particularly dynamic and reversible protein post-translational modification (PTM) that occurs extensively in both histone and non-histone proteins in eukaryotic and prokaryotic organisms^1–8^. Kac proteins are capable of regulating the expression of defense-related genes and various enzyme activities. They function as components in protein complexes^9–13^ involved in defense and stress responses, signal transduction, energy and material metabolism, and virulence^14–22^.

In recent years, Kac proteins and their functions have received much attention. Advances in proteomic technology based on ultra-high performance liquid chromatography (UPLC), tandem mass spectrometry (LC–MS/MS), and acetylation-specific antibody enrichment have together identified a large number of Kac sites and proteins, providing useful information on biological processes and mechanisms^23–28^. In filamentous fungi, Kac events are closely related to various cellular activities and pathogenicity. Deletion of *MoHat1,* an encoding histone acetyltransferase, in *Magnaporthe oryzae* resulted in a significant decrease in growth, conidiation, and virulence toward rice and barley^29^. MoHat1 can regulate acetylation of proteins related to appressorium formation and pathogenicity of *M. oryzae*^30^. In *Fusarium graminearum*, 577 Kac sites have been identified, of which 10 were involved in deoxynivalenol (vomitoxin, DON) biosynthesis and fungal virulence^31^. In *Beauveria bassiana*, 283 Kac proteins and 464 Kac sites have been identified, and mutation of the Kac sites of mannosyltransferases Pmt1 and Pmt4 attenuated virulence toward *Galleria mellonella*, and decreased tolerance to oxidation stress and cell wall perturbation^32^.

*Clonostachys chloroleuca* (formerly *Clonostachys rosea*) 67–1 is an important mycoparasite and has shown excellent biocontrol effects on various plant fungal diseases, such as cucumber Fusarium wilt and rice sheath blight^33,34^; however, the functions of Kac events in this biocontrol fungus remain unclear. Analysis of the transcriptome of *C. chloroleuca* 67–1 during mycoparasitism of *S. sclerotiorum* sclerotia identified a number of genes encoding acetyltransferase and deacetylase enzymes that were significantly differentially expressed^35^, suggesting that Kac events might be involved in 67–1 mycoparasitism. The acetylation may affect the biological activities of the biocontrol fungus by regulating the expression of mycoparasitism-related genes and/or influencing the activities of proteins that contribute to signal transduction, defense responses and mycoparasitic processes^28,31^. The proteomic strategies have been established to yield sound data in plant pathogenic fungi *Phytophthora sojae* and *B. cinerea* in our previous studies^36,37^, which is adapted to the beneficial fungus *C. chloroleuca*. In this research, the lysine acetylome of strain 67–1 during mycoparasitism to sclerotia was constructed, and Kac proteins and sites were identified and characterized. The results provide a comprehensive view of the molecular mechanisms regulated by Kac events in the biocontrol activity of *C. chloroleuca* against plant fungal pathogens.

## Results

### Identification and characterization of Kac proteins and sites in *C. chloroleuca* 67–1

**Figure 1:**
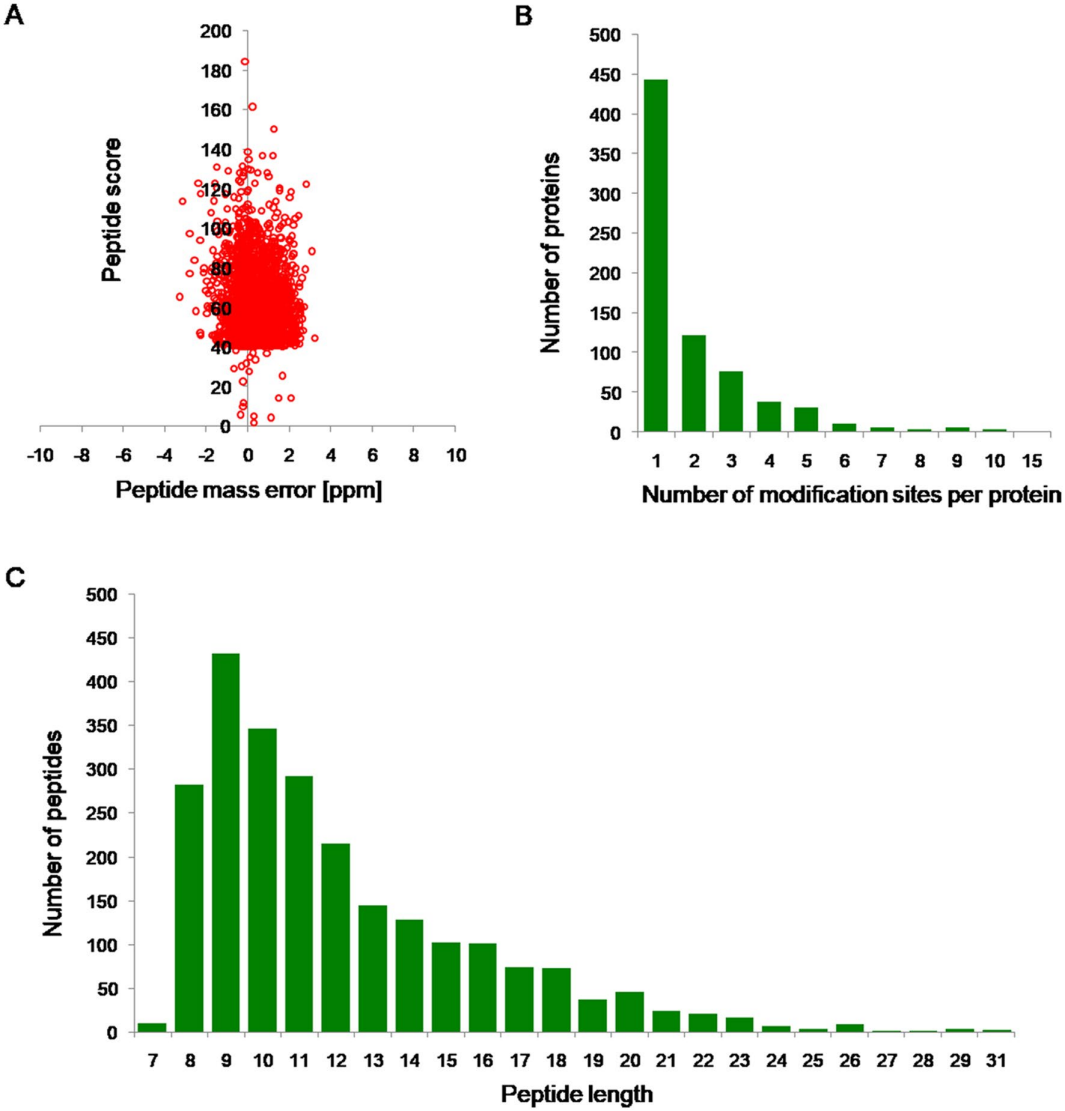
Properties of the identified peptides in *C. chloroleuca* 67–1. (**A**) Distribution of peptide mass errors. (**B**) Distribution of lysine acetylation (Kac) sites. (**C**) Length of Kac peptides.

The Kac modification of 67–1 during mycoparasitic process and vegetative growth were conducted. The mycelia of 67–1 collected in different infection stages were combined as one sample to reflect the mycoparasitic process of *C. chloroleuca*, while the mycelia without the induction of sclerotia served as a control. A quantitative lysine acetylome of *C. chloroleuca* 67–1 was generated by TMT labelling, affinity purification, and LC–MS/MS. The results of repeatability tests showed that the quantitative data were statistically consistent (Fig. S1). Mass errors of most Kac peptides were within 3 ppm, which is consistent with precise MS analysis. The peptides ranged in length from 8 to 18 amino acids (Fig. 1A, C), which is consistent with the expected fragments for trypsin-based enzymatic hydrolysis of protein samples. The proportion of Kac proteins containing 1 − 3 Kac sites was 86.5%, while 13.5% of proteins contained more than 3 Kac sites, giving an average of 1.8 Kac sites per protein (Fig. 1B). A total of 1448 Kac sites on 740 proteins were identified (Table S1)**,** accounting for 15% of all proteins in *C. chloroleuca* 67–1. The acetylome results indicated that Kac events may play important roles in regulating *C. chloroleuca* mycoparasitism.

### Analysis of Kac motifs in *C. chloroleuca*

**Figure 2:**
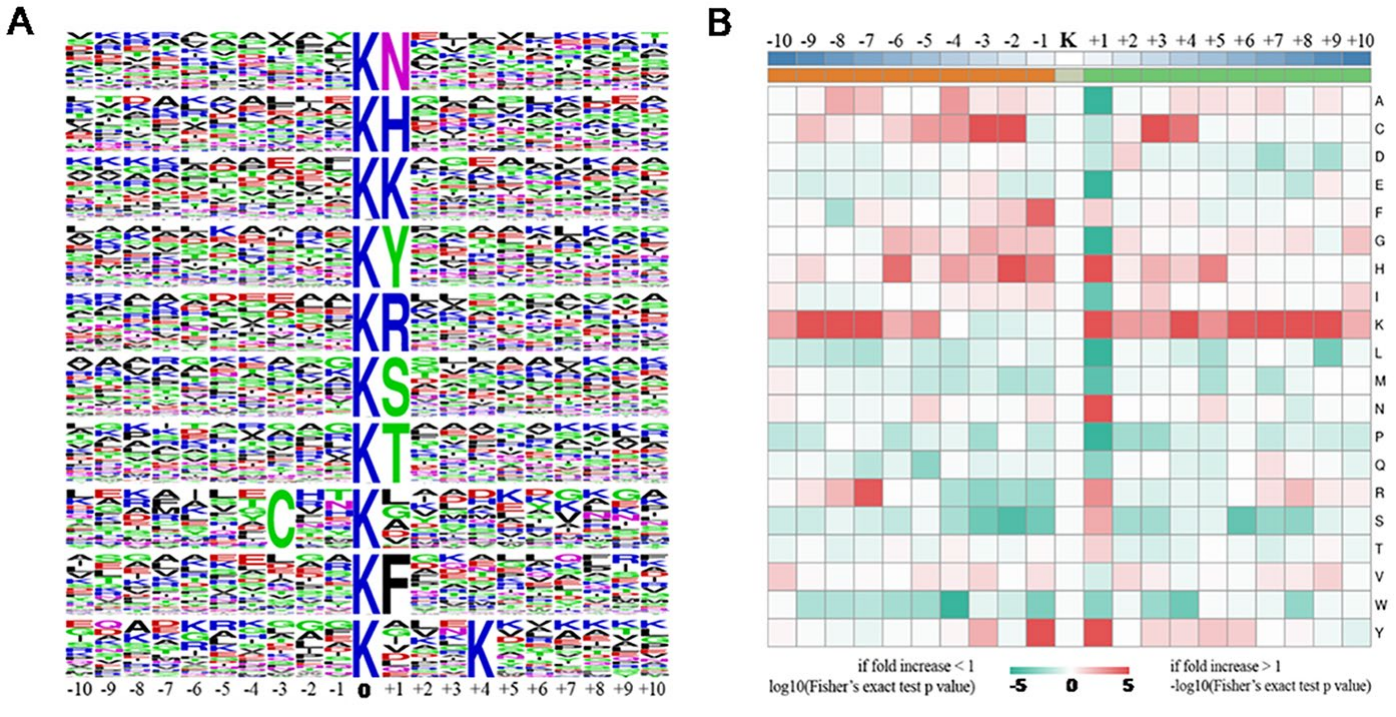
Motif analysis of identified Kac sites in *C. chloroleuca* 67–1. (**A**) Kac motifs and conservation of Kac sites. The size of each letter corresponds to the frequency of the amino acid residues in a given position. (**B**) Heatmap of the amino acid composition of Kac sites.

To further investigate the characteristics of acetylation sites in *C. chloroleuca* 67–1 during the mycoparasitic process, conserved sequence motifs in the 1431 identified peptides were evaluated, revealing 10 conserved sequences surrounding Kac sites. Most of the conserved residues are located downstream of Kac sites, with asparagine (N), histidine (H), lysine (K), tyrosine (Y), arginine (R), serine (S), threonine (T) and phenylalanine (F) in the + 1 position, lysine (K) in the + 4 position, and cystine (C) conserved upstream in the -3 position (Fig. 2, Table S2). Among the 10 motifs, four are highly conserved in both eukaryotic and prokaryotic organisms. Among the remaining six motifs of KacK, KacN, KacR, KacS, KacT and CxxKac, KacS was only detected in the acetylomes of *Trichinella sprialis* and *Aspergillus flavus*, while KacN, KacK, KacR and KacT were only reported in *T. sprialis*^38,39^ in previous analyses of plant pathogens and biocontrol fungi. The CxxKac motif appears to be unique to *C. chloroleuca*, detected herein at 26 Kac sites on 25 Kac proteins associated with binding domains of pyridoxal-5’-phosphate (PLP)-dependent transferases and histidine kinases involved in oxytetracycline biosynthesis and cytokinin activities; however, most of these proteins have not yet been characterized. The results indicate that some Kac events are of special importance for fungi, and more Kac proteins and sites are required to be explored in future work.

### Characteristics of differentially regulated Kac sites and proteins in *C. chloroleuca*

**Figure 3:**
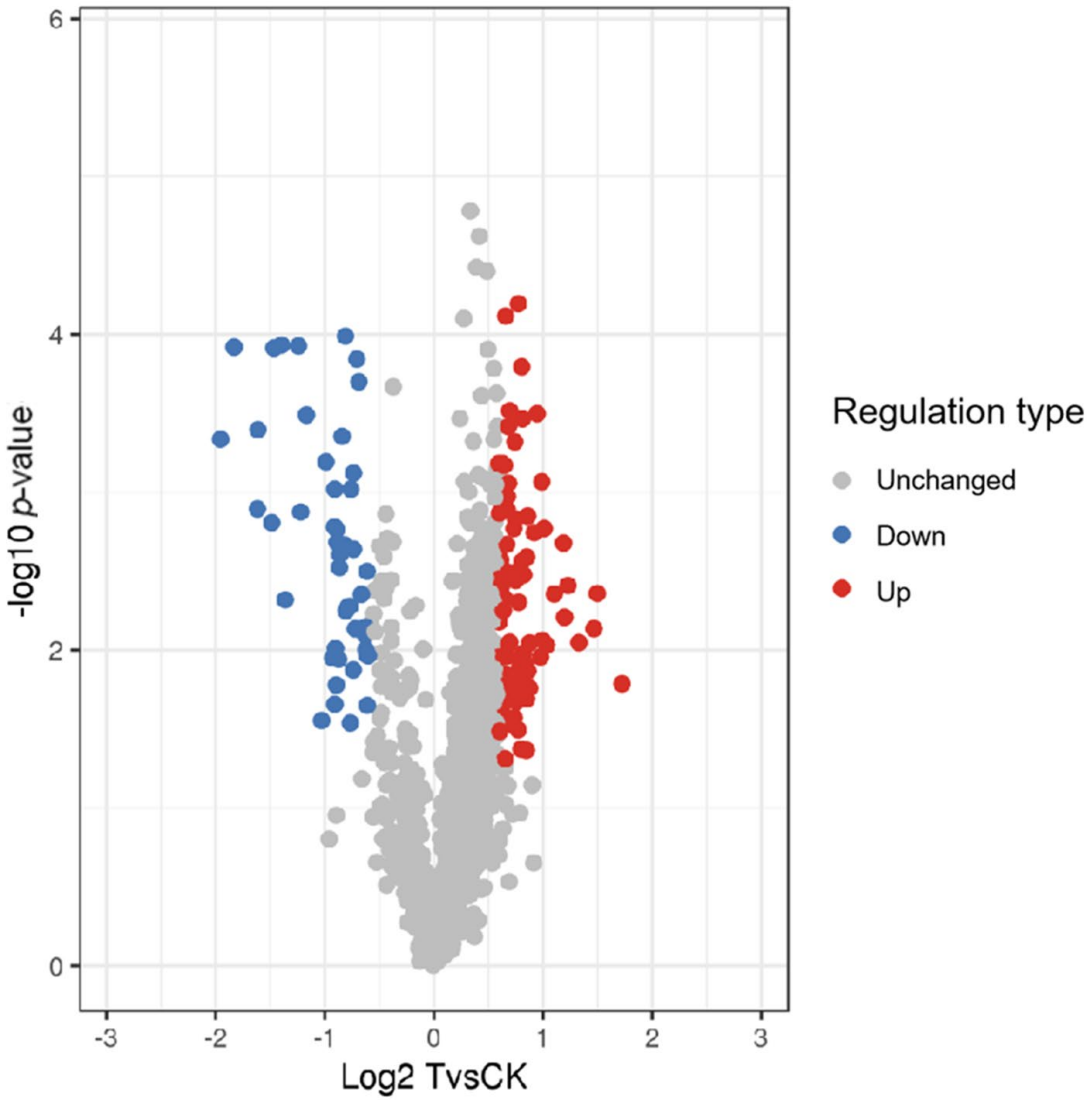
Volcano plot of differentially modified Kac sites in *C. chloroleuca* 67–1.

**Figure 4:**
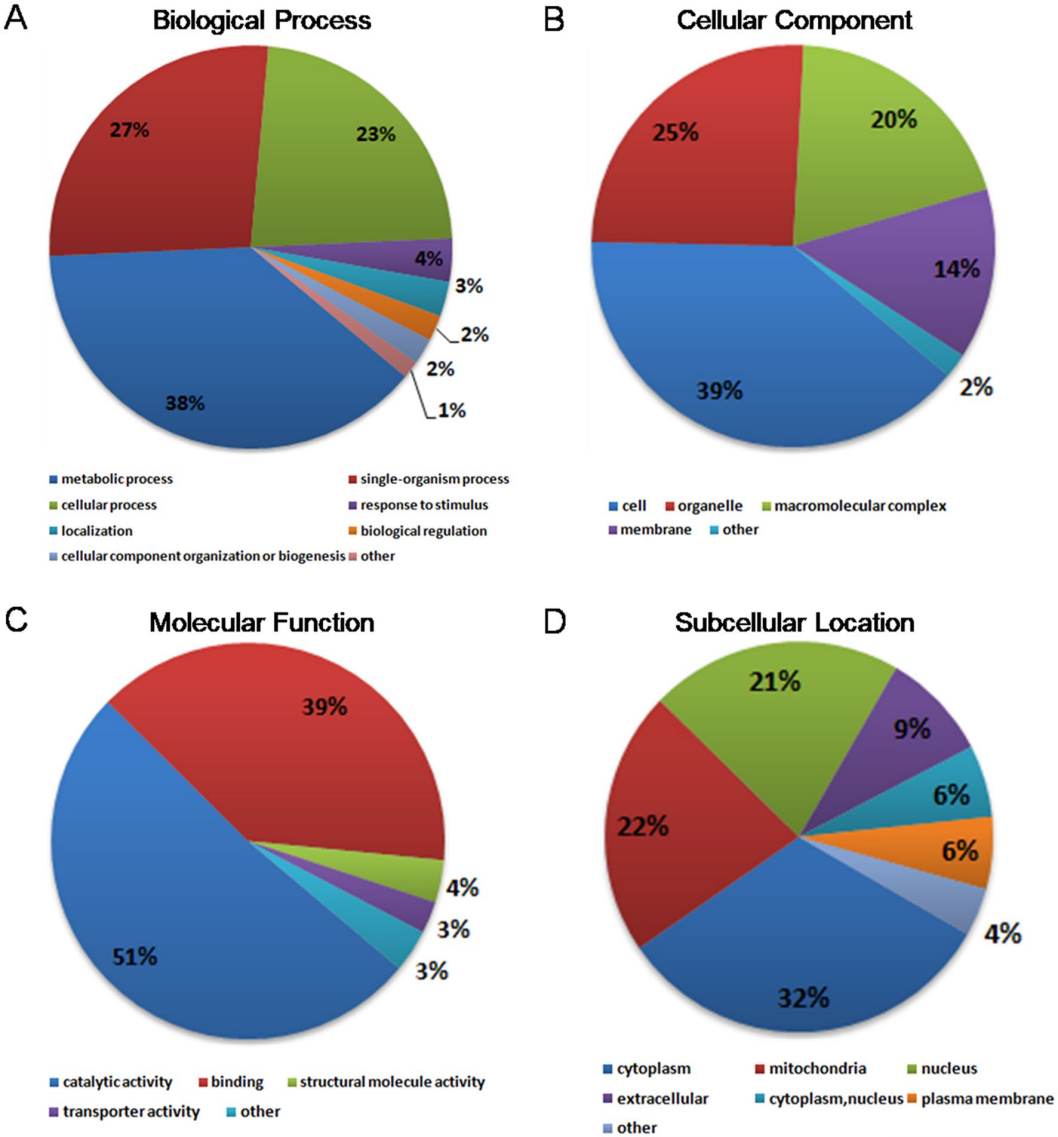
GO functional distribution and subcellular localization of differentially regulated Kac proteins in *C. chloroleuca* 67–1. (**A**) Biological process classification. (**B**) Cellular component classification. (**C**) Molecular function classification. (**D**) Subcellular localization.

Among the quantified acetylated proteins, 80 Kac sites were up-regulated and 46 sites were down-regulated during the mycoparasitic process in *C. chloroleuca* 67–1, compared with vegetative growth (*P* < 0.05, Fig. 3, Table S3). Gene Ontology (GO) analysis of the three main functional categories (biological process, molecular function and cellular component) was performed. In the biological process category, 38% of differentially regulated Kac proteins were associated with metabolic process, 27% were related to single-organism process, and 23% were linked to cellular process. Additionally, proteins were found to be involved in responses, localization and biological regulation (Fig. 4A, Table S4). In the cellular component category, 39% of the proteins were associated with the cell wall and cell envelope, 25% were linked to organelles, 20% were related to macromolecular complexes, and 14% were membrane-associated (Fig. 4B). In the molecular function category, catalytic activity and binding activity were the most important processes, accounting for 90% of the identified differentially regulated Kac proteins (Fig. 4C). Analysis of the subcellular localization of the differentially regulated acetylated proteins in *C. chloroleuca* during sclerotia induction showed that most proteins were located in the cytoplasm (32%), followed by the mitochondria (22%) and the nucleus (21%), while 9% were found to be extracellular (Fig. 4D, Table S5).

**Figure 5:**
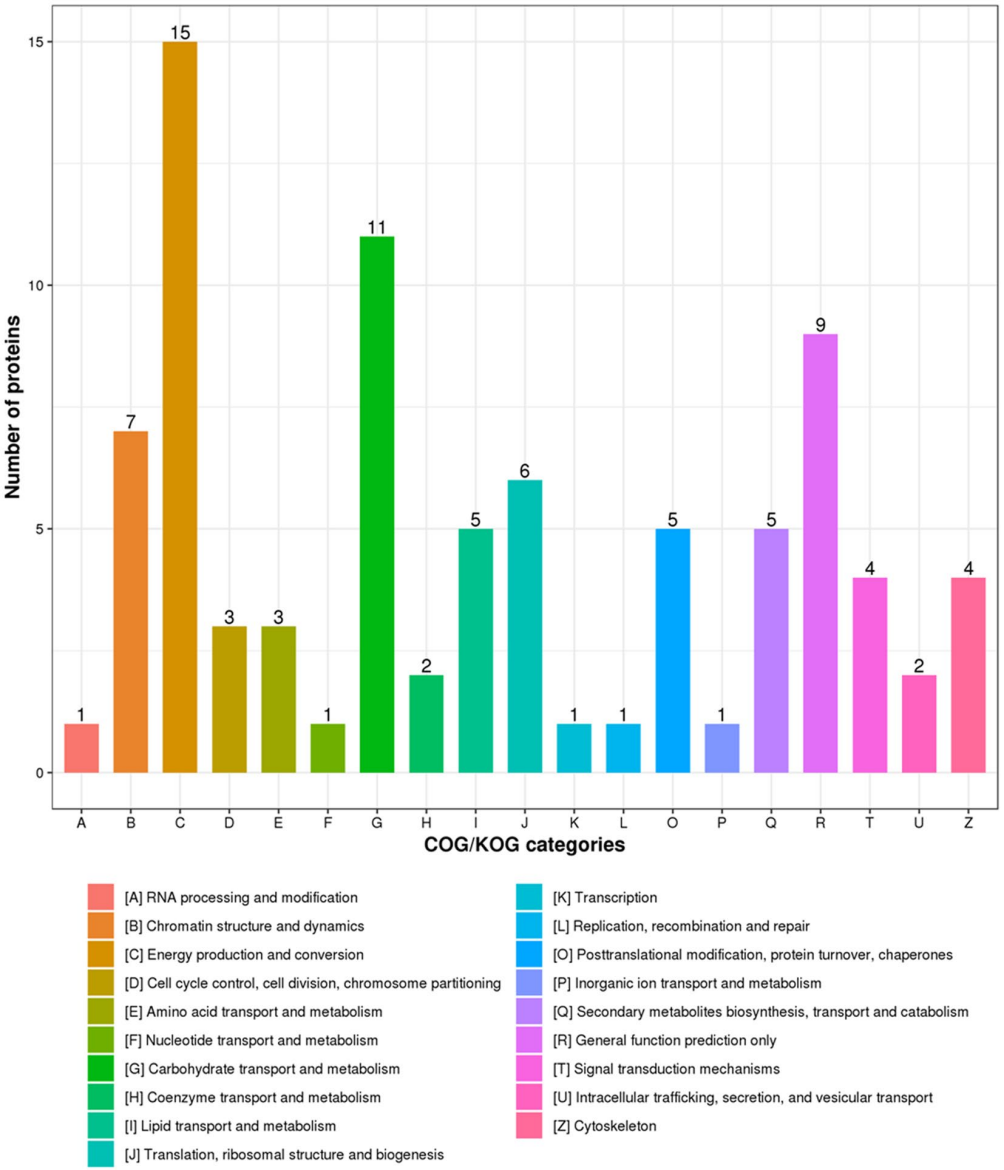
KOG functional classification of differentially regulated Kac proteins in *C. chloroleuca* 67–1.

In Eukaryotic Orthologous Group (KOG) classification, ~ 70% of differentially regulated acetylated proteins were related to metabolism and cellular processes such as carbohydrate transport and metabolism, and energy production and conversion. However, the functions of 10.9% of the modified proteins were not clear (Fig. 5, Table S6). The results of GO, KOG and subcellular localization analyses were consistent; differentially regulated Kac sites and proteins are involved in diverse functions during *C. chloroleuca* mycoparasitism, especially metabolism, oxidation–reduction processes, and binding.

### *C. chloroleuca* enrichment analysis

**Figure 6:**
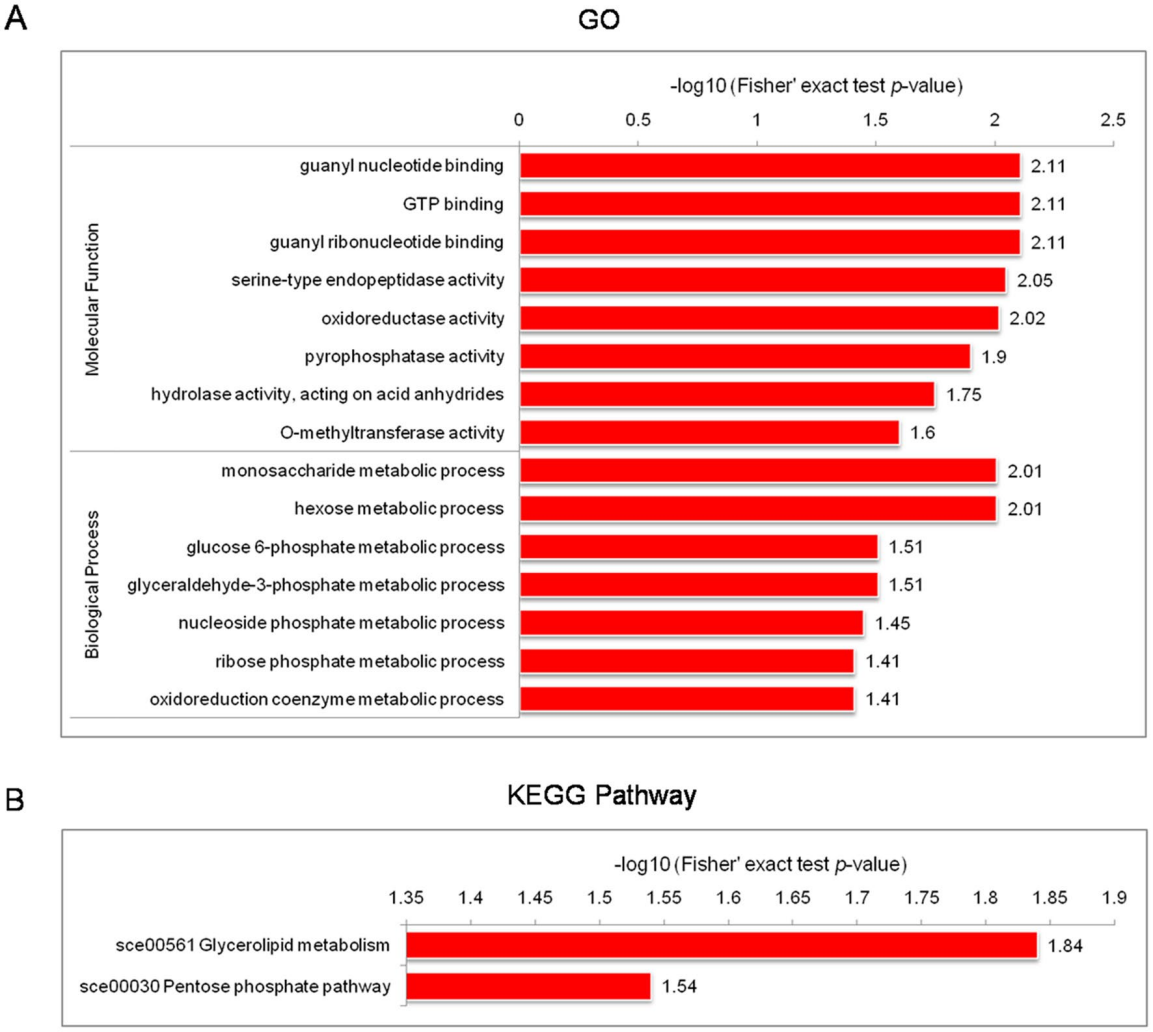
Enrichment analysis of up-regulated Kac proteins in *C. chloroleuca* 67–1. (**A**) GO-based enrichment analysis. (**B**) KEGG pathway-based enrichment analysis.

To detect the enrichment trends of the differentially regulated Kac sites and proteins, GO and Kyoto Encyclopedia of Genes and Genomes (KEGG) pathway enrichment analyses were performed. GO analysis showed that up-regulated proteins were strongly linked to binding activities, multiple metabolism processes, and oxidoreductase activities, such as GTP binding and metabolism of monosaccharides (especially hexose and ribose phosphate), and many function as components of enzyme complexes (Fig. 6A, Table S7). In these pathways, guanine nucleotide-binding protein, glucose-6-phosphate isomerase (PGI), triose-phosphate isomerase (TPI), glyceraldehyde-3-phosphate dehydrogenase (GAPD), 6-phosphogluconate dehydrogenase (G6PD), catalase/peroxidase HPI, cytochrome C oxidase, cytochrome P450, and chitinase were markedly enriched. By contrast, Kac proteins associated with dimerization, chromatin assembly, and nucleosome organization were significantly down-regulated, especially histone 2A variant and histone H3. Dihydrolipoamide succinyltransferase (DLST) and dihydrolipoyl dehydrogenase (DLDH), involved in the glyoxylate cycle, were also markedly down-regulated (Table S8).

**Figure 7:**
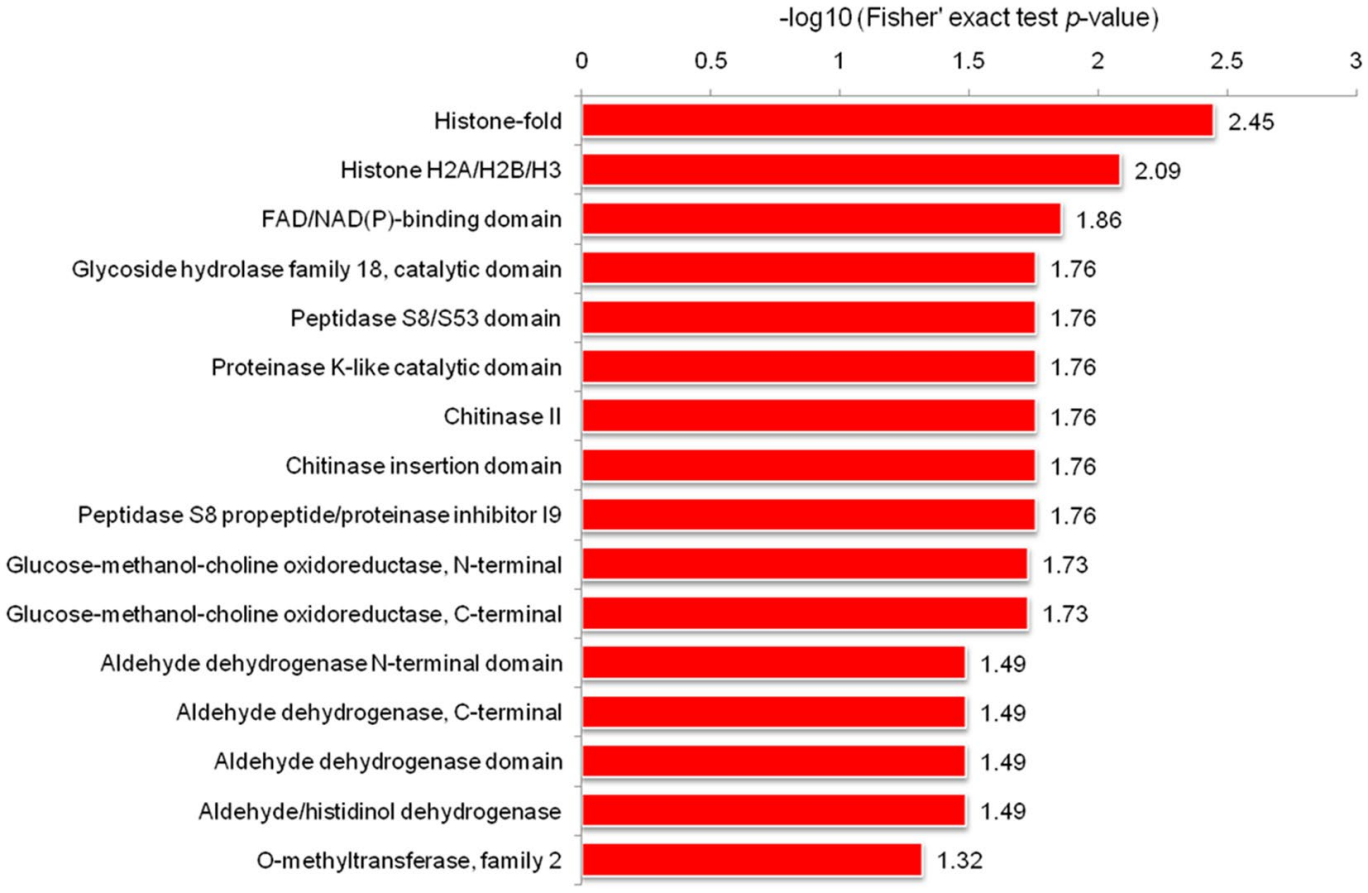
Domain-based enrichment analysis of all differentially regulated Kac proteins in *C. chloroleuca* 67–1.

KEGG pathway enrichment analysis indicated that many up-regulated Kac proteins were associated with glycerolipid metabolism and the pentose-phosphate pathway (PPP) (Fig. 6B), and the down-regulated proteins were consistent with those identified by GO enrichment analysis (Table S8). In addition, the identified Kac protein domains were predicted to be histones, peptidases, oxidoreductases, aldehyde dehydrogenase (ALDH), and FAD/NAD(P)-binding domains (Fig. 7), all closely related to various cellular activities based on GO and KEGG enrichment analyses. In general, Kac events are active during chromatin assembly, energy metabolism, the tricarboxylic acid cycle (TCA) and glycometabolism in *C. chloroleuca* induced by *S. sclerotiorum*.

### *C. chloroleuca* protein interaction network analysis

**Figure 8:**
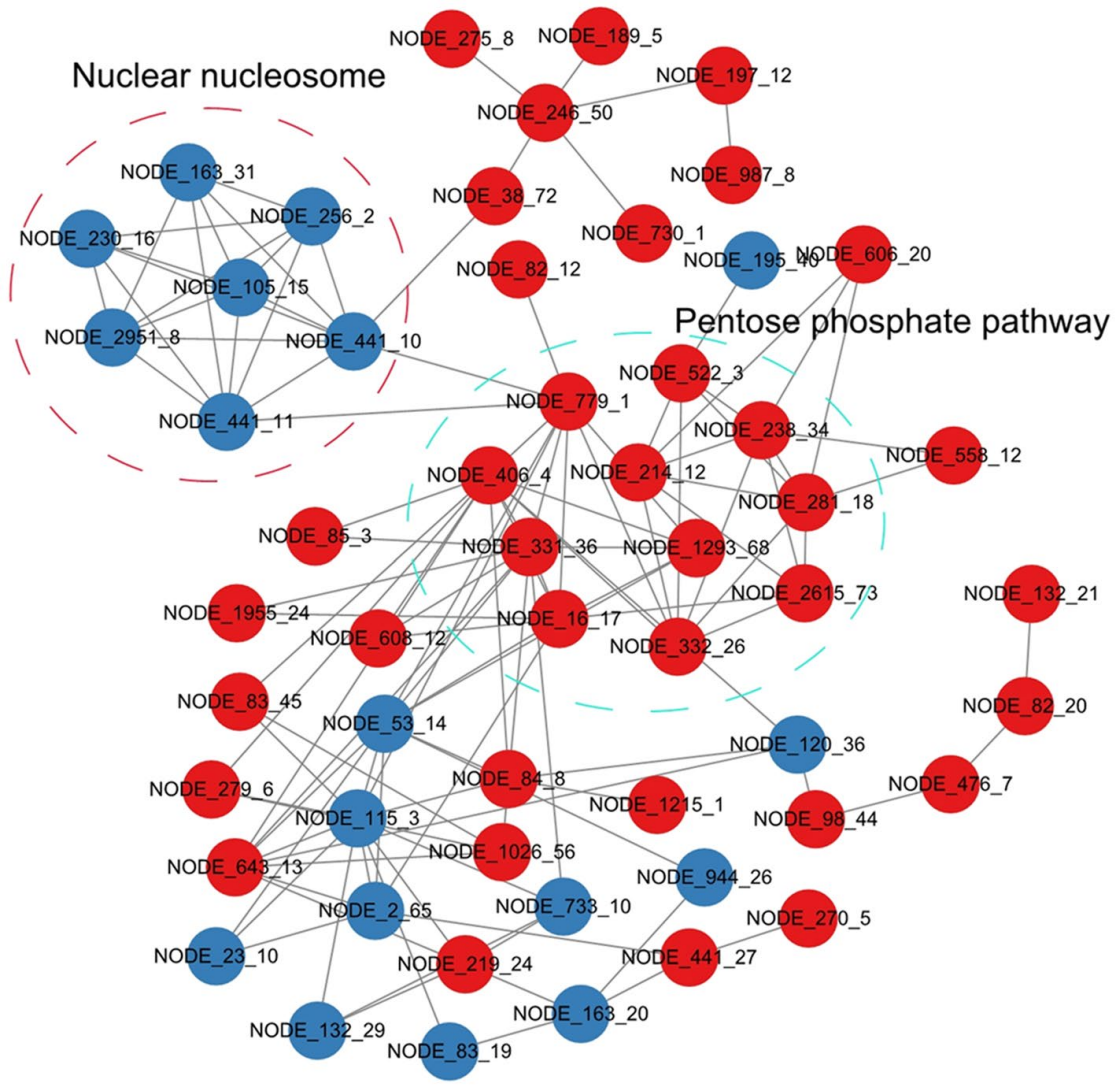
Protein–protein interaction network of differentially regulated modification sites in *C. chloroleuca* 67–1.

A protein interaction network was established using STRING. Two groups comprising 11 and 7 proteins were associated with the nuclear nucleosome pathway and the PPP, respectively (Fig. 8, Table S9). These results further confirm our conclusion that Kac proteins associated with chromatin assembly and glycometabolism are essential during *C. chloroleuca* mycoparasitism.

## Discussion

Although Kac is a widespread and highly conserved PTM of proteins in all organisms, its functions have not been extensively explored in filamentous fungi^37^. In the current study, 740 Kac proteins were identified, which accounting for 15% of all proteins in *C. chloroleuca* 67–1, and the proportion of Kac proteins identified was much higher than previously reported for *P. sojae*, *B. cinerea*, *F. graminearum* and *B. bassiana*^31,32,35,36^. To the best of our knowledge, this is the first report on the correlation between Kac events and the biocontrol activity of *C. chloroleuca*. The findings reveal the functions of Kac proteins in *C. chloroleuca* mycoparasitism, and provide insight into the molecular mechanisms underlying the biological control activity of the fungus against plant fungal pathogens.

As an important mycoparasite, *C. chloroleuca* has great potential for controlling a range of plant fungal diseases under various environmental conditions^35^. Many research efforts have been made in the understanding of its mycoparasitic strategies. It is well known that ATP and NADPH are produced in the PPP, and NADPH has reducing power for anabolism and maintains the redox balance of cells, while intermediate products are used for biosynthesis^40^. Therefore, we believe that acetylation of proteins involved in carbohydrate metabolism and energy production and conversion may be essential during *C. chloroleuca* mycoparasitism, and we speculate that the expression levels of catalase/peroxidase enzymes are differentially up-regulated in the mycoparasite during the response to stimulation of *S. sclerotiorum*. In addition, the glyoxylate cycle, which complements the TCA, can increase the utilization of acetyl-CoA and the production of succinic acid to boost the energy supply. Additional experiments will be needed to conclusively prove these findings.

Consistent with GO and KEGG enrichment analyses of the Kac proteins, the domains enrichment analysis demonstrated that these proteins were more predicted to be histones, peptidases, oxidoreductases, ALDH and FAD/NAD(P)-binding domains. Previous studies proposed that histone deacetylases utilizing NAD^+^ as a cofactor are sensitive to nutrient levels in cells^40^. When energy is limited, NAD^+^ levels increase and histone deacetylases are activated, and a series of metabolic signals are transduced by deacetylation^17,40,41^. We speculate that Kac events may monitor the intracellular nutrient and energy status during *C. chloroleuca* vegetative growth and mycoparasitic process. In addition, NAD^+^ is also a coenzyme of dehydrogenases, and it is essential in glycolysis, gluconeogenesis, the TCA, and the respiratory chain. All these findings strongly suggest that lysine acetylation is important for biological control activity of *C. chloroleuca* against plant fungal pathogens.

In conclusion, these findings represent the first extensive data on lysine acetylation in *C. chloroleuca*. These data not only indicate that the regulatory scope of lysine acetylation is broad in *C. chloroleuca*, but also expands our current knowledge of the molecular mechanisms underlying *C. chloroleuca* control of plant fungal pathogens regulated by Kac proteins.

## Methods

### Strains

*C. chloroleuca* 67–1 (ACCC 39,160) was originally isolated from a vegetable garden in Hainan Province, China. *S. sclerotiorum* Ss-H (ACCC 39,161) was isolated from an infected soybean plant in a field in Heilongjiang Province, China. Both isolates were cultured on potato dextrose agar (PDA) medium at 26 °C and maintained at 4 °C. All strains and plants were obtained with the permission of relevant departments and the study was in compliance with local and national regulations.

### Preparation of *C. chloroleuca* 67–1 protein samples

A 100 μL volume of 10^7^ spores/mL 67–1 suspension was smeared evenly on a PDA plate (ϕ 90 mm). After incubation at 26 °C for 48 h, a wafer-thin layer of mycelia appeared, and dozens of uniformly sized and surface-sterilized sclerotia of *S. sclerotiorum* were laid out compactly onto the surface of the 67–1 plates. The sclerotia were removed at 8, 24 and 48 h after encountering, and the mycelia of *C. chloroleuca* cultures at different timepoints were collected and mixed as one sample. Plates of 67–1 without sclerotia served as a control, experiments were conducted three times, and a total of six samples were frozen immediately at -80 °C in liquid nitrogen^35^.

Mycelia from *C. chloroleuca* were ground to powder in liquid nitrogen using a mortar and pestle, and transferred to 2 mL tubes containing lysis buffer with 10 mM dithiothreitol, 1% protease inhibitor cocktail, 3 μM trichostatin A (TSA) and 50 mM nicotinamide (NAM). The samples were ultrasonicated on ice using a High-intensity Ultrasound Processor (Scientz, Ningbo, China). An equal volume of Tris-saturated phenol (pH 8.0) was added and mixed by vortexing for 5 min. The mixture was centrifuged at 5 000 g at 4 °C for 10 min, and the upper phenol phase was transferred into a new tube containing four volumes of ammonium sulphate-saturated methanol. The samples were incubated at − 20 °C for 6 h then centrifuged at 4 °C for 10 min. The precipitated proteins were collected and washed with ice-cold methanol followed by three washes with ice-cold acetone. The proteins were re-dissolve in 8 M urea and determined using a BCA Protein Assay Kit (Beyotime, Shanghai, China).

### Trypsin digestion of *C. chloroleuca* 67–1 protein samples

The protein samples were reduced with dithiothreitol at a final concentration of 5 mM at 56 °C for 30 min, then alkylated with iodoacetamide at a final concentration of 11 mM at room temperature in darkness for 15 min. Trypsin was added at a ratio of 1:50 (trypsin/protein, w/w) and incubated overnight, and 1:100 trypsin was then added and incubated for 4 h to thoroughly digest protein samples.

### TMT labelling

The peptides were desalted using a Strata X C18 SPE Column (Phenomenex, Torrance, CA, USA), vacuum-dried, and reconstituted in 0.5 M triethylammonium bicarbonate (TEAB). The samples were labelled using a TMT 6-plex Labelling Kit (Thermo Fisher Scientific, Rockford, IL, USA) according to the manufacturer’s instructions.

### HPLC fractionation

The labelled peptides were eluted with a gradient of 8−32% acetonitrile (ACN) using a Thermo Betasil C18 Column (Thermo Fisher Scientific). A total of 60 fractions were collected per min by reversed-phase HPLC, mixed into four groups, and dried using a vacuum freeze centrifuge (Eppendorf, Hamburg, Germany).

### Affinity enrichment of Kac peptides

The tryptic peptides were dissolved in IP buffer (100 mM NaCl, 1 mM EDTA, 50 mM Tris–HCl, 0.5% NP-40, pH 8.0) and incubated with pre-washed PTM104 antibody beads (PTM Bio, Hangzhou, China) at 4 °C with gentle shaking. After 12 h, beads were removed, washed with IP buffer four times and ultrapure H_2_O twice, and bound peptides were eluted with 0.1% trifluoroacetic acid (TFA) three times, then desalted using C18 ZipTips (Millipore, Shanghai, China) and vacuum-dried.

### LC–MS/MS analysis

The peptides were dissolved in Solvent A containing 0.1% formic acid (FA) and 2% ACN, and loaded onto a reversed-phase analytical column on an EASY-nLC 1000 UPLC System (Agilent, Santa Clara, CA, USA). For gradient elution, the amount of solvent B (0.1% FA and 90% ACN) was increased from 8 to 20% over 19 min, from 20 to 32% over 3 min, and increased to 80% over 4 min, all at a constant flow rate of 320 nL/min.

The peptides were injected into a nanospray ionization (NSI) source and quantified by LC–MS/MS using a Q Exactive Plus instrument (Thermo Scientific, San Jose, CA, USA) coupled online to the UPLC. For full MS analysis, the scan ranged from 350 to 1800 m*/z* and the mass resolution in the orbitrap was set at 70,000. For HCD MS/MS analysis, the scan range was fixed at 100 m*/z* and the mass resolution was 17,500. A data-dependent procedure was employed by alternating one MS scan and 20 MS/MS scans with 15 s dynamic exclusion and 30% high-energy C-trap dissociation (HCD) at an electrospray voltage of 2.0 kV. Automatic gain control was set at 5E4, the threshold ion count was 5000 ions/s, and the maximum injection time was 200 ms.

### Quantitative analysis of differentially modified proteins

The influence of protein abundance on the modified signals was eliminated by quantitative proteome normalization, the fold-change for differential modification of Kac sites under different treatments was calculated, and the *p*-value of the differential modification ratio was determined by *t*-test; proteins with *p* < 0.05 and fold-change > 1.5 were considered significantly up-regulated, while those with fold-change < 1/1.5 were considered significantly down-regulated. A volcano plot was drawn in which the horizontal axis represents multiple values for protein differences after Log2 conversion and the vertical axis represents the *p*-value after transformation of -log10 for significance of difference tests.

### Analysis of the repeatability of quantitative data

To analyze the data derived from three repeated experiments, principal component analysis (PCA), relative standard deviation (RSD) and Pearson’s correlation coefficient were used, and modified quantitative repeatability was evaluated.

### Database searching

The resulting LC–MS/MS data were searched against the *C. chloroleuca* 67–1 mycoparasitism-related gene database^35^ concatenated with the reverse decoy database using Maxquant (v1.5.2.8), and a common reference database was appended to eliminate the effects of potential contaminants. Trypsin/P was specified as the cleavage enzyme, up to four missed cleavages were allowed, the minimal peptide length was set at 7 residues, and maximal number of modification sites per peptide was set at 5. The mass tolerance for precursor ions was set at 20 ppm and 5 ppm for First search and Main search, respectively, and for fragment ions it was set at 0.02 Da. Carbamidomethyl on Cys was selected as a fixed modification, while oxidation on Met, acetylation on Lys and acetylation on the protein N-terminus were selected as variable modifications. TMT 6-plex was selected as the quantitative method. The false discovery rate (FDR) thresholds for proteins, peptides and modification sites were adjusted at 1%, and the site localization probability was set to no less than 0.75.

### Bioinformatics analysis

The Kac proteins with differentially modified sites were analyzed using multiple bioinformatic tools. GO analysis was performed for functional classification and enrichment with the UniProt-GOA database (http://www.ebi.ac.uk/GOA/). InterProScan and InterPro (http://www.ebi.ac.uk/interpro/) were used to classify and enrich Kac protein domains, respectively, and InterProScan was also used to analyze proteins unannotated by GO. WoLFPSORT (http://wolfpsort.seq.cbrc.jp/) was used to predict the subcellular localization of Kac proteins. The differentially modified proteins were mapped using KOG analysis (http://genome.jgi.doe.gov/help/ kogbrowser.jsf), and functional pathways of the Kac proteins were annotated and enriched using KEGG analysis (http://www.genome.jp/kegg/). All protein sequences with differential modifications were searched against STRING (version 10.5) for protein–protein interactions, and interactions with high confidence scores (> 0.7) were retained. The top 50 Kac proteins with the closest interactions were selected for graph-theoretical clustering algorithm and molecular complex detection (MCODE) analysis. The online software Motif-x (http://motif-x.med.harvard.edu/) was used to predict the motif sequences of all identified Kac positions.

In these analyses, all database protein sequences were used as the background database parameter, and other parameters were set to default values. For each category, a two-tailed Fisher’s exact test was employed to assess the proportions of differentially modified proteins in each protein annotation category. Any annotation categories with Fisher’s exact test *p*-values < 0.05 were considered strongly enriched.

## Supplementary Information

Supplementary Information 1.

Supplementary Information 2.

Supplementary Information 3.

Supplementary Information 4.

Supplementary Information 5.

Supplementary Information 6.

Supplementary Information 7.

Supplementary Information 8.

Supplementary Information 9.

Supplementary Information 10.
