# Supplementary material for: Tandem Mass Tag labelling quantitative acetylome analysis of differentially modified proteins during mycoparasitism of Clonostachys chloroleuca 67–1
Source: Sci Rep. 2021 Nov 17;11:22383. doi: 10.1038/s41598-021-01956-2 (PMC8599485; doi:10.1038/s41598-021-01956-2)

**Figure S1. Sample repeatability tests.** (A) PCA distribution of all samples. (B) RSD distribution of repeated samples. (C) Heatmap of Pearson correlation coefficients from all quantified proteins between each pair of samples.


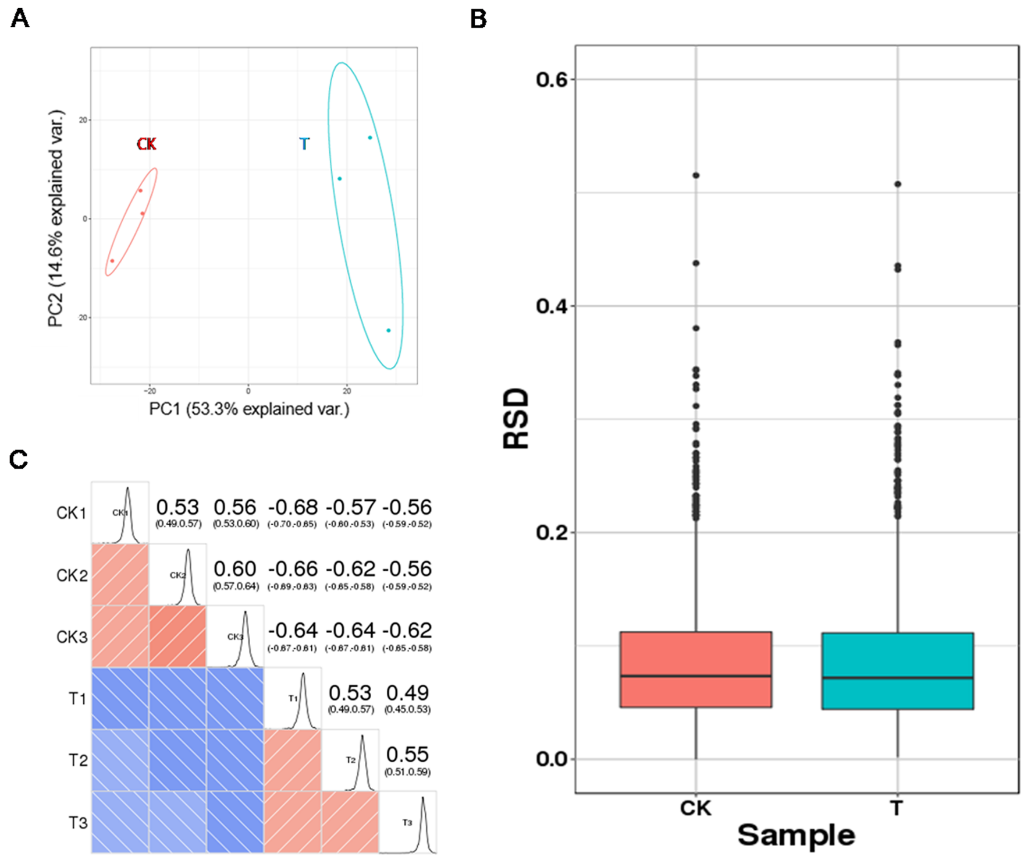

Supplement: Supplementary file 1 — Supplementary Information 1. [file 41598_2021_1956_MOESM1_ESM.docx]
