# Supplementary material for: Tandem Mass Tag labelling quantitative acetylome analysis of differentially modified proteins during mycoparasitism of Clonostachys chloroleuca 67–1
Source: Sci Rep. 2021 Nov 17;11:22383. doi: 10.1038/s41598-021-01956-2 (PMC8599485; doi:10.1038/s41598-021-01956-2)
Supplement: Supplementary file 2 — Supplementary Information 2. [file 41598_2021_1956_MOESM2_ESM.pdf]

Table S1. Modification Sites Identified in the *C. rosea* 67-1 Acetylome

protein accession	position	protein description	score	charge	mass error [ppm]
NODE_100_5	147	hypothetical protein	93.649	3	-1.8548
NODE_100_5	93	hypothetical protein	61.167	4	4.3551
NODE_100_5	182	hypothetical protein	78.884	3	-0.74846
NODE_100_5	75	hypothetical protein	46.318	5	-0.82645
NODE_100_5	229	hypothetical protein	49.448	3	-1.3664
NODE_1018_4	88	predicted protein	67.207	2	-2.1531
NODE_1018_4	17	predicted protein	40.475	3	-0.47746
NODE_1018_4	78	predicted protein	52.716	2	0.43246
NODE_101_28	451	uncharacterized protein	59.153	3	0.16265
NODE_1026_56	126	nadp-dependent alcohol dehydrogenase	53.775	3	0.15869
NODE_1035_3	1010	hypothetical protein	57.525	3	2.5127
NODE_1035_7	457	NADH-ubiquinone oxidoreductase 51 kDa subunit	57.985	3	2.6012
NODE_1035_7	395	NADH-ubiquinone oxidoreductase 51 kDa subunit	43.502	3	-1.4849
NODE_1035_7	307	NADH-ubiquinone oxidoreductase 51 kDa subunit	59.367	3	0.049388
NODE_1035_9	168	predicted protein	51.286	2	-0.76582
NODE_1037_14	58	related to Y.lipolytica GPR1 protein and Fun34p	43.03	3	1.0877
NODE_1037_39	692	peptidase M1 family protein	77.221	3	0.46011
NODE_1037_39	657	peptidase M1 family protein	67.08	2	-1.5583
NODE_1037_41	34	PLP-dependent transferase	55.724	4	0.094313
NODE_1037_53	59	VPS29-involved in vacuolar protein sorting	43.308	3	-0.42874
NODE_1038_11	69	hypothetical protein	90.601	2	-1.0245
NODE_1038_11	64	hypothetical protein	64.121	3	0.40827
NODE_1038_11	108	hypothetical protein	59.265	3	0.87368
NODE_1038_20	69	hypothetical protein	59.067	3	-2.9411
NODE_1039_2	14	hypothetical protein	119.25	2	-0.91547
NODE_105_15	131	hypothetical protein	84.479	2	-1.4541
NODE_105_15	90	hypothetical protein	69.03	2	-2.252
NODE_105_15	9	hypothetical protein	120.23	3	-0.29091
NODE_105_15	14	hypothetical protein	120.23	3	-0.73429
NODE_105_15	20	hypothetical protein	120.23	3	-0.73429
NODE_105_15	37	hypothetical protein	65.224	2	-2.8905
NODE_105_15	45	hypothetical protein	72.34	2	-0.42425
NODE_105_15	24	hypothetical protein	47.712	3	-0.6552
NODE_105_15	30	hypothetical protein	95.477	2	-1.4277
NODE_105_15	29	hypothetical protein	85.536	2	-0.27973
NODE_105_16	6	histone H2A	56.548	2	-1.0931
NODE_105_16	10	histone H2A	69.331	2	-1.0931
NODE_105_16	15	histone H2A	122.18	3	0.19887
NODE_105_26	1075	hypothetical protein	56.916	4	-1.0609
NODE_1085_3	527	2-oxoglutarate dehydrogenase E1 component	41.513	3	-1.6113
NODE_1085_3	629	2-oxoglutarate dehydrogenase E1 component	57.484	5	-0.59776
NODE_1085_3	1003	2-oxoglutarate dehydrogenase E1 component	82.241	3	-1.842
NODE_108_3	455	uncharacterized protein	53.756	4	0.83935
NODE_1095_38	78	hypothetical protein	63.419	3	-0.39018
NODE_1095_38	133	hypothetical protein	51.643	5	0.20831
NODE_110_32	152	Trehalose-phosphatase	51.949	3	-0.6886
NODE_110_42	73	ras gtpase	123.95	2	0.64322
NODE_110_46	92	glutamine synthetase	81.017	2	1.036
NODE_110_46	241	glutamine synthetase	55.98	2	-0.87514
NODE_110_46	248	glutamine synthetase	94.309	4	-2.328
NODE_110_46	301	glutamine synthetase	49.298	3	1.1101
NODE_111_15	154	putative transesterase protein	46.892	3	0.53349
NODE_1125_1	94	hypothetical protein	40.186	3	3.6568

NODE_112_14	1176	oxidosqualene:lanosterol cyclase	101.56	3	3.736
NODE_112_23	37	carboxylic acid transport protein	75.738	3	-0.20668
NODE_113_10	117	uncharacterized protein	93.345	2	1.6369
NODE_113_12	652	S-adenosylmethionine-dependent methyltransferase superfamily domain-containing protein	51.276	3	2.0473
NODE_113_17	467	hypothetical protein	87.639	3	-0.65491
NODE_113_17	502	hypothetical protein	66.022	3	-0.77377
NODE_113_17	111	hypothetical protein	82.417	3	-0.060195
NODE_113_17	300	hypothetical protein	86.898	3	-0.28332
NODE_113_41	93	hypothetical protein	44.543	3	1.5936
NODE_1152_4	149	uncharacterized protein	92.265	3	-0.99826
NODE_115_13	327	prolidase pepP, putative	77.062	3	-0.98301
NODE_115_15	63	uncharacterized protein	45.368	3	-1.4722
NODE_115_15	311	uncharacterized protein	44.612	2	2.3769
NODE_115_18	17	hypothetical protein	52.247	3	-0.6107
NODE_115_3	1896	predicted protein	49.298	3	-2.8442
NODE_115_3	1465	predicted protein	51.726	3	-1.4021
NODE_117_13	189	yeats-domain-containing protein	101.38	2	0.42229
NODE_117_13	191	yeats-domain-containing protein	101.38	2	0.42229
NODE_117_13	186	yeats-domain-containing protein	101.38	2	0.42229
NODE_117_13	184	yeats-domain-containing protein	78.078	4	-0.11711
NODE_117_15	108	NAD dependent epimerase/dehydratase family protein	113.43	2	-1.6356
NODE_119_36	144	uncharacterized protein	45.829	2	-0.69995
NODE_11_14	356	signal transducing adaptor molecule	79.474	2	0.050749
NODE_11_8	446	putative translation release factor erf3	64.121	3	-0.91053
NODE_120_13	68	hypothetical protein	83.862	2	0.51944
NODE_120_13	100	hypothetical protein	41.399	3	0.922
NODE_120_15	64	hypothetical protein	55.806	2	1.4587
NODE_120_26	91	integral membrane protein	122.69	3	0.21417
NODE_120_33	123	uricase	62.617	5	0.31535
NODE_120_33	204	uricase	54.982	2	-0.81632
NODE_120_33	91	uricase	100.93	5	-0.49357
NODE_120_36	251	probable NDE1-mitochondrial cytosolically directed NADH dehydrogenase	50.305	3	-2.055
NODE_1215_1	843	NRPS-like enzyme, putative	42.314	3	-1.3581
NODE_1215_1	584	NRPS-like enzyme, putative	66.267	4	0.01946
NODE_1215_1	300	NRPS-like enzyme, putative	54.982	3	-0.42642
NODE_121_9	177	uncharacterized protein	77.597	2	0.71146
NODE_123_18	45	hypothetical protein	43.635	3	-0.78293
NODE_123_18	61	hypothetical protein	127.83	4	0.16189
NODE_123_5	329	hypothetical protein	68.536	3	0.47382
NODE_125_43	97	putative mitochondrial phosphate carrier protein	51.066	3	-2.4032
NODE_125_47	160	hypothetical protein	52.527	3	-0.92412
NODE_125_66	576	Calpain-9	47.991	3	4.3689
NODE_126_34	638	hypothetical protein	56.432	3	1.4353
NODE_126_48	271	Oxysterol-binding protein	59.067	3	-1.4228
NODE_126_48	142	Oxysterol-binding protein	77.42	3	-0.44364
NODE_126_49	327	fructose-bisphosphate aldolase, class II	72.433	3	-1.8894
NODE_126_49	359	fructose-bisphosphate aldolase, class II	45.433	2	-1.6236
NODE_126_49	244	fructose-bisphosphate aldolase, class II	77.185	5	-1.2069
NODE_126_49	10	fructose-bisphosphate aldolase, class II	83.692	2	-0.24389
NODE_126_49	114	fructose-bisphosphate aldolase, class II	102.89	3	-1.1233
NODE_126_55	128	double-stranded RNA-binding type zinc finger domain protein	49.3	2	0.54302
NODE_127_22	32	hypothetical protein	44.468	3	4.2278
NODE_127_50	451	WD repeat protein	61.582	2	-1.8294

NODE_1289_5	343	isoamyl alcohol oxidase	40.798	4	-0.59449
NODE_1291_57	107	hypothetical protein	46.069	3	-0.59115
NODE_1293_68	685	glucose-6-phosphate isomerase	78.342	2	0.042638
NODE_1293_68	972	glucose-6-phosphate isomerase	89.189	3	-0.51871
NODE_1293_68	1125	glucose-6-phosphate isomerase	77.185	3	-0.25965
NODE_12_21	105	probable ribosomal protein RPL4A	43.308	2	-3.2471
NODE_12_21	184	probable ribosomal protein RPL4A	59.198	2	-1.3595
NODE_12_25	309	predicted protein	74.841	2	-0.04694
NODE_12_26	48	hypothetical protein	96.342	3	0.096718
NODE_12_34	124	NADP-specific glutamate dehydrogenase	98.055	2	0.89418
NODE_12_36	272	vacuolar sorting protein 1	53.136	3	2.8915
NODE_130_17	253	hypothetical protein	45.115	3	-0.39291
NODE_130_24	242	aldo/keto reductase	83.948	2	-1.4068
NODE_130_53	625	6-phosphofructokinase-like protein	78.903	3	0.039358
NODE_130_70	338	elongation factor 3	52.247	3	-1.4709
NODE_132_14	702	predicted protein	51.726	4	-0.55649
NODE_132_14	1461	predicted protein	48.284	3	-2.06
NODE_132_14	693	predicted protein	77.74	3	-1.2269
NODE_132_21	427	predicted protein	108.56	2	-0.59535
NODE_132_21	447	predicted protein	46.797	4	-0.078555
NODE_132_21	502	predicted protein	102.21	4	2.0846
NODE_132_21	388	predicted protein	66.073	3	-0.28284
NODE_132_26	401	hypothetical protein	46.069	3	-0.23603
NODE_132_26	70	hypothetical protein	60.55	3	-0.63215
NODE_132_26	458	hypothetical protein	46.89	3	-0.24833
NODE_132_26	66	hypothetical protein	49.3	3	1.2889
NODE_132_26	195	hypothetical protein	61.962	2	-1.41
NODE_132_27	145	hypothetical protein	58.981	3	-1.1074
NODE_132_29	551	hypothetical protein	108.14	3	1.1449
NODE_132_8	30	hypothetical protein	44.71	3	4.2342
NODE_1343_7	38	putative cell division control protein 11 protein	59.728	3	-1.2036
NODE_1343_7	199	putative cell division control protein 11 protein	40.449	3	-0.66061
NODE_136_7	80	glutathione reductase	86.794	3	-0.9489
NODE_1376_2	265	hypothetical protein	66.989	3	0.023313
NODE_13_31	250	hypothetical protein	55.314	3	-0.49531
NODE_13_31	594	hypothetical protein	58.172	2	-0.5415
NODE_13_31	496	hypothetical protein	83.862	2	-1.4684
NODE_1400_8	193	putative serine/threonine phosphatase 2C ptc2	70.028	2	-1.26
NODE_140_3	139	hypothetical protein	56.205	2	-1.2054
NODE_140_5	80	hypothetical protein	93.325	2	-0.40912
NODE_141_13	309	isocitrate dehydrogenase subunit 2 precursor	47.971	3	-0.14314
NODE_141_13	160	isocitrate dehydrogenase subunit 2 precursor	40.724	3	-0.44502
NODE_141_13	311	isocitrate dehydrogenase subunit 2 precursor	51.03	3	-2.5099
NODE_141_3	227	reptin	49.448	3	0.78298
NODE_141_6	479	uncharacterized protein	49.3	2	-1.0996
NODE_142_12	64	catalase-peroxidase	45.28	3	-1.3301
NODE_142_12	424	catalase-peroxidase	82.452	3	0.03083
NODE_142_12	381	catalase-peroxidase	43.592	3	-0.96045
NODE_143_16	175	hypothetical protein	61.942	3	-1.9877
NODE_147_1	207	predicted protein	56.404	3	-1.9455
NODE_147_1	364	predicted protein	55.064	2	-0.54464
NODE_147_1	82	predicted protein	83.206	2	0.67108
NODE_147_10	398	tubulin alpha chain	51.113	3	-0.30839
NODE_147_10	53	tubulin alpha chain	53.444	3	-2.1186
NODE_147_10	329	tubulin alpha chain	64.121	2	-0.61555
NODE_147_10	105	tubulin alpha chain	45.384	3	0.5056

NODE_147_10	331	tubulin alpha chain	60.489	3	-2.1869
NODE_148_25	281	hypothetical protein	66.056	2	1.7963
NODE_148_25	279	hypothetical protei	44.925	3	0.38405
NODE_14_11	436	sodium transport ATPase	44.511	4	-0.82635
NODE_14_9	344	hypothetical protein	90.861	3	-0.96874
NODE_1506_2	350	putative cytochrome p450 protein	49.076	4	0.60431
NODE_1506_2	93	putative cytochrome p450 protein	100.02	2	-1.3998
NODE_1506_2	218	putative cytochrome p450 protein	85.29	3	0.38135
NODE_1506_2	247	putative cytochrome p450 protein	43.68	3	0.50918
NODE_150_1	163	hypothetical protein	59.198	3	0.39172
NODE_1511_126	301	tryptophanyl-tRNA synthetase	63.816	3	-1.4437
NODE_1511_140	170	hypothetical protein	65.719	3	-1.8695
NODE_1511_140	138	hypothetical protein	54.259	2	-0.7205
NODE_1511_147	244	probable beta (1-3) glucanosyltransferase gel3p	65.184	3	-1.7539
NODE_1511_147	70	probable beta (1-3) glucanosyltransferase gel3p	53.448	3	0.56887
NODE_1511_147	284	probable beta (1-3) glucanosyltransferase gel3p	40.493	3	3.2503
NODE_1511_147	425	probable beta (1-3) glucanosyltransferase gel3p	45.433	2	-0.75075
NODE_1511_153	126	ATP-binding cassette sub-family F member 2	46.318	3	-0.67083
NODE_1511_171	85	predicted protein	47.088	3	4.281
NODE_1511_171	488	predicted protein	61.161	2	-0.45793
NODE_1511_171	194	predicted protein	74.464	2	-0.61643
NODE_1511_171	607	predicted protein	47.187	3	1.4948
NODE_1511_171	480	predicted protein	42.083	3	-1.3021
NODE_1511_171	614	predicted protein	51.643	3	-3.4968
NODE_1511_171	622	predicted protein	91.867	3	-0.95414
NODE_1511_171	465	predicted protein	69.979	4	-2.1237
NODE_1511_171	331	predicted protein	57.164	3	-0.24085
NODE_1511_171	445	predicted protein	97.384	3	-1.6719
NODE_1511_171	551	predicted protein	65.179	2	-2.9918
NODE_1511_171	566	predicted protein	57.288	2	-1.4636
NODE_1511_171	383	predicted protein	78.342	2	-0.66516
NODE_1511_171	43	predicted protein	47.621	3	0.1867
NODE_1511_171	557	predicted protein	59.908	3	-0.23104
NODE_1511_33	268	dihydroxy-acid dehydratase	85.536	3	-0.98007
NODE_1527_13	233	formyl transferase	46.68	3	3.4324
NODE_1531_23	103	arf gtpase-activating protein	52.247	2	-1.0136
NODE_1531_9	14	hypothetical protein	66.267	3	3.4324
NODE_154_33	625	hypothetical protein	57.288	3	-1.2562
NODE_155_11	231	AhpC-TSA-domain-containing protein	70.056	2	-0.43482
NODE_155_11	171	AhpC-TSA-domain-containing protein	93.096	2	1.0782
NODE_155_13	10	hypothetical protein	41.399	3	-2.7797
NODE_155_13	79	hypothetical protein	61.11	4	1.0955
NODE_155_16	170	cellular nucleic acid-binding protein	42.314	3	3.5912
NODE_155_26	91	probable SONA	60.764	4	0.11193
NODE_155_36	294	hypothetical protein	42.149	4	-3.3811
NODE_155_36	386	hypothetical protein	46.069	3	1.4789
NODE_1585_1	69	predicted protein	84.653	4	-1.3629
NODE_1585_1	232	predicted protein	84.365	2	-0.33564
NODE_1585_1	160	predicted protein	58.123	3	0.089563
NODE_1590_3	477	predicted protein	50.353	3	-2.1533
NODE_1598_1	972	Myosin	53.237	2	-0.54832
NODE_15_11	210	hypothetical protein	75.548	3	-0.26866
NODE_15_2	216	hypothetical protein	72.315	3	-0.71109
NODE_15_2	299	hypothetical protein	82.261	3	1.103
NODE_15_2	257	hypothetical protein	80.236	3	3.6266
NODE_15_25	244	40S ribosomal protein S4	54.259	2	-0.81518

NODE_15_25	53	40S ribosomal protein S4	59.35	2	-1.483
NODE_15_25	37	40S ribosomal protein S4	104.01	3	0.93237
NODE_15_37	44	redoxin	105.46	3	0.48787
NODE_15_49	166	hypothetical protein	89.355	3	1.2586
NODE_15_49	57	hypothetical protein	57.175	2	-1.3939
NODE_15_49	168	hypothetical protein	61.165	3	-0.66018
NODE_15_49	247	hypothetical protein	77.324	3	-0.3543
NODE_161_68	78	guanylate kinase	83.137	3	1.0264
NODE_161_89	488	predicted protein	58.511	3	-1.7771
NODE_162_3	353	hypothetical protein	85.813	2	-0.76473
NODE_162_3	360	hypothetical protein	76.478	3	0.89732
NODE_162_8	18	hypothetical protein	54.023	3	-0.2016
NODE_162_8	283	hypothetical protein	41.242	3	0.50514
NODE_162_8	70	hypothetical protein	70.555	3	-1.4708
NODE_162_8	285	hypothetical protein	57.347	2	-0.62561
NODE_163_20	197	Isocitrate lyase	73.834	4	-1.5124
NODE_163_20	332	Isocitrate lyase	60.019	2	1.4295
NODE_163_20	322	Isocitrate lyase	72.891	2	-0.81256
NODE_163_24	371	predicted protein	73.834	3	0.34302
NODE_163_24	383	predicted protein	66.893	3	2.3547
NODE_163_24	335	predicted protein	49.3	3	-1.2179
NODE_163_26	278	ATPase get3	46.069	2	-1.0484
NODE_163_31	354	hypothetical protein	72.434	4	-0.30593
NODE_163_31	469	hypothetical protein	45.28	3	-2.0919
NODE_165_6	405	hypothetical protein	49.536	3	0.52008
NODE_16_12	163	predicted protein	61.11	3	-0.78617
NODE_16_12	150	predicted protein	57.106	3	-1.6164
NODE_16_17	345	probable transaldolase	53.168	2	-0.62855
NODE_16_17	124	probable transaldolase	67.334	2	0.92455
NODE_16_17	123	probable transaldolase	68.224	2	-1.1876
NODE_16_17	254	probable transaldolase	93.374	3	-3.5728
NODE_16_17	302	probable transaldolase	70.134	3	-0.13474
NODE_16_17	228	probable transaldolase	58.699	3	-0.10889
NODE_16_17	160	probable transaldolase	62.287	3	0.15034
NODE_16_17	68	probable transaldolase	41.513	3	0.13522
NODE_16_17	316	probable transaldolase	86.911	2	-0.80329
NODE_16_25	96	uncharacterized protein	89.355	3	0.093975
NODE_16_7	284	hypothetical protein	43.798	2	-1.9744
NODE_16_7	400	hypothetical protein	63.611	3	-0.51001
NODE_16_7	394	hypothetical protein	47.712	2	1.3969
NODE_171_23	35	hypothetical protein	44.625	4	-0.38336
NODE_171_27	38	hypothetical protein	49.765	4	2.56
NODE_171_28	63	60S acidic ribosomal protein P0	44.925	3	-0.037118
NODE_171_28	7	60S acidic ribosomal protein P0	65.627	2	0.54968
NODE_171_30	564	glycoside hydrolase family 38	55.755	3	0.49157
NODE_171_30	536	glycoside hydrolase family 38	83.647	3	-2.0306
NODE_171_43	278	ATP-dependent RNA helicase eIF4A	82.417	2	-0.099539
NODE_171_7	349	DnaJ domain-containing protein	42.336	4	-0.072477
NODE_171_7	264	DnaJ domain-containing protein	99.283	2	0.27502
NODE_171_7	364	DnaJ domain-containing protein	45.022	2	-0.13125
NODE_173_6	387	sterigmatocystin 8-o-methyltransferase	64.82	2	-1.6642
NODE_173_6	298	sterigmatocystin 8-o-methyltransferase	51.286	4	-2.1099
NODE_173_6	91	sterigmatocystin 8-o-methyltransferase	46.796	3	0.17835
NODE_173_6	48	sterigmatocystin 8-o-methyltransferase	82.452	3	-0.33696
NODE_1758_1	124	chitinase	47.227	3	-0.035072
NODE_1758_3	152	ubiquitin/small ribosomal subunit protein 31 fusion protein	71.451	2	-1.5588

NODE_1758_3	104	ubiquitin/small ribosomal subunit protein 31 fusion protein	85.212	2	-0.94592
NODE_1758_3	6	ubiquitin/small ribosomal subunit protein 31 fusion protein	80.69	2	-1.695
NODE_1758_3	48	ubiquitin/small ribosomal subunit protein 31 fusion protein	72.29	3	-1.3202
NODE_176_16	72	probable hydroxymethy glutaryl-CoA synthase	42.843	2	-0.77707
NODE_176_16	310	probable hydroxymethyl glutaryl-CoA synthase	67.019	2	-0.6055
NODE_177_24	59	hypothetical protein	76.326	3	0.97842
NODE_177_24	53	hypothetical protein	46.462	2	-1.4539
NODE_177_24	270	hypothetical protein	57.047	3	-1.238
NODE_17_145	122	uncharacterized protein	63.216	3	-0.34884
NODE_17_159	121	hypothetical protein	40.994	3	1.3106
NODE_17_25	382	N-acetyl-beta-D-glucosaminidase [*Clonostachys rosea*]	73.26	4	0.25345
NODE_17_28	894	uncharacterized protein	51.469	4	3.343
NODE_17_28	825	uncharacterized protein	56.036	3	-1.4045
NODE_180_11	516	hypothetical protein	65.295	2	0.22781
NODE_180_11	536	hypothetical protein	70.056	3	-0.37497
NODE_180_11	326	hypothetical protein	57.267	3	0.84199
NODE_180_15	838	putative leucyl-trna synthetase protein	41.117	3	1.1474
NODE_180_17	35	hypothetical protein	60.118	3	-0.33586
NODE_180_17	74	hypothetical protein	54.764	4	-0.69051
NODE_180_17	87	hypothetical protein	42.785	3	-0.86626
NODE_180_17	62	hypothetical protein	82.452	2	0.70614
NODE_180_34	98	predicted protein	49.768	3	2.8784
NODE_181_135	861	predicted protein	76.064	2	1.4193
NODE_181_135	226	predicted protein	55.531	2	-0.86259
NODE_181_142	167	aspartyl-tRNA synthetase	71.085	3	-1.4272
NODE_181_175	294	glycosyltransferase family 20 protein	50.353	2	-0.95722
NODE_181_175	264	glycosyltransferase family 20 protein	52.579	2	0.38448
NODE_181_185	4	proteasome regulatory subunit	55.353	3	1.3925
NODE_181_39	136	FAD binding domain protein	52.576	3	1.2593
NODE_184_31	466	translocation protein SEC63	51.591	3	-3.8308
NODE_185_13	613	hypothetical protein	45.68	3	0.37147
NODE_1861_8	12	uncharacterized protein	65.224	2	0.63335
NODE_189_13	1489	hypothetical protein	63.419	3	-0.49042
NODE_189_20	315	GFA1-glucosamine-fructose-6-phosphate transaminase	61.577	2	0.069602
NODE_189_20	339	GFA1-glucosamine-fructose-6-phosphate transaminase	52.579	3	-1.8565
NODE_189_20	547	GFA1-glucosamine-fructose-6-phosphate transaminase	74.789	3	0.5794
NODE_189_20	125	GFA1-glucosamine-fructose-6-phosphate transaminase	42.718	3	0.15736
NODE_189_20	54	GFA1-glucosamine-fructose-6-phosphate transaminase	83.182	3	0.55895
NODE_189_20	47	GFA1-glucosamine-fructose-6-phosphate transaminase	54.898	3	-0.39611
NODE_189_5	421	AGC/NDR/NDR protein kinase	45.876	4	2.9388
NODE_18_5	224	hypothetical protein	53.998	4	1.8477
NODE_191_40	600	adenylosuccinate lyase	42.031	3	2.1536
NODE_192_74	467	Pc21g17770	73.057	4	1.2998
NODE_1955_10	90	hypothetical protein	46.063	3	-0.27138
NODE_1955_132	194	DNA glycosylase	77.662	3	0.0016509
NODE_1955_141	399	mitochondrial cytochrome b2	61.11	3	1.5403
NODE_1955_141	374	mitochondrial cytochrome b2	54.259	2	-1.0884
NODE_1955_154	673	dipeptidyl-peptidase III	64.654	3	-0.23292

NODE_1955_154	83	dipeptidyl-peptidase III	44.318	3	3.7996
NODE_1955_154	188	dipeptidyl-peptidase III	51.566	3	-0.37587
NODE_1955_155	118	hypothetical protein	73.499	2	-0.083119
NODE_1955_155	36	hypothetical protein	43.761	3	0.025994
NODE_1955_155	117	hypothetical protein	67.385	3	-0.92013
NODE_1955_155	167	hypothetical protein	71.176	2	-1.1115
NODE_1955_155	126	hypothetical protein	57.973	3	-0.30579
NODE_1955_159	106	endosomal P24B protein	40.067	5	2.8457
NODE_1955_164	49	hypothetical protein PFICI_01218	69.261	3	-0.040076
NODE_1955_24	472	6-phosphogluconate dehydrogenase, decarboxylating 2	48.527	2	0.037191
NODE_1955_24	84	6-phosphogluconate dehydrogenase,decarboxylating 2	50.108	3	-0.53233
NODE_1955_24	501	6-phosphogluconate dehydrogenase, decarboxylating 2	58.373	4	2.3202
NODE_1955_24	155	6-phosphogluconate dehydrogenase, decarboxylating 2	66.962	3	2.2749
NODE_1955_24	292	6-phosphogluconate dehydrogenase, decarboxylating 2	48.423	2	-0.039562
NODE_1955_24	329	6-phosphogluconate dehydrogenase, decarboxylating 2	52.579	2	-0.88449
NODE_1955_24	347	6-phosphogluconate dehydrogenase, decarboxylating 2	46.334	2	-0.31366
NODE_1955_24	410	6-phosphogluconate dehydrogenase, decarboxylating 2	80.96	3	-1.0982
NODE_1955_39	427	hypothetical protein	88.495	3	0.58647
NODE_1955_39	545	hypothetical protein	56.404	2	-2.5589
NODE_1955_43	35	GTP-binding nuclear protein GSP1/Ran	66.692	3	-1.0925
NODE_1955_43	21	GTP-binding nuclear protein GSP1/Ran	52.527	3	-0.40688
NODE_1955_43	132	GTP-binding nuclear protein GSP1/Ran	65.224	3	-1.6513
NODE_1955_43	97	GTP-binding nuclear protein GSP1/Ran	46.955	3	-1.19
NODE_1955_43	140	GTP-binding nuclear protein GSP1/Ran	89.301	3	-0.05447
NODE_1955_43	121	GTP-binding nuclear protein GSP1/Ran	59.728	4	-1.526
NODE_1955_74	304	ATP synthase alpha chain,mitochondrial precursor	52.172	3	-0.695
NODE_1955_74	217	ATP synthase alpha chain,mitochondrial precursor	70.197	3	0.20494
NODE_1955_74	239	ATP synthase alpha chain,mitochondrial precursor	64.121	2	-0.58349
NODE_1955_74	471	ATP synthase alpha chain, mitochondrial precursor	76.064	3	-1.2098
NODE_1955_74	160	ATP synthase alpha chain, mitochondrial precursor	43.592	3	-0.60976
NODE_1955_74	315	ATP synthase alpha chain, mitochondrial precursor	82.651	3	-0.39945
NODE_1955_74	164	ATP synthase alpha chain, mitochondrial precursor	76.943	2	0.31383
NODE_1955_74	229	ATP synthase alpha chain,mitochondrial precursor	129.76	2	-0.20577
NODE_1955_74	423	ATP synthase alpha chain, mitochondrial precursor	138.45	2	0.22292
NODE_1955_94	38	hypothetical protein	54.259	3	0.49178
NODE_195_40	19	hypothetical protein	67.334	3	0.77168
NODE_195_5	142	26S proteasome subunit P45 family protein	43.68	3	3.4068
NODE_195_5	106	26S proteasome subunit P45 family protein	59.35	2	0.070007
NODE_197_1	987	Phosphoribosylformyl glycinamidine synthase	53.597	3	-0.26801
NODE_197_12	237	predicted protein	83.456	2	-2.6603
NODE_197_12	161	predicted protein	87.99	4	-0.65646
NODE_197_12	265	predicted protein	52.866	3	-1.8403
NODE_197_12	83	predicted protein	77.662	2	-0.99366
NODE_197_12	186	predicted protein	86.772	2	-1.1232
NODE_197_6	367	hypothetical protein	60.255	3	-0.56287
NODE_197_6	195	hypothetical protein	50.353	2	0.91063
NODE_198_17	244	translation initiation factor eIF-2B subunit alpha	42.708	3	3.6579
NODE_198_41	705	hypothetical protein	89.484	4	-0.45272
NODE_198_44	281	hypothetical protein	67.334	2	-1.97

NODE_198_66	155	phosphatidylinositol transporter, putative	42.633	3	2.9127
NODE_198_7	544	hypothetical protein	71.03	3	2.8136
NODE_198_7	1170	hypothetical protein	78.342	4	0.15378
NODE_199_13	216	40S ribosomal protein S2	63.662	2	-1.3734
NODE_199_9	67	hypothetical protein	57.859	2	0.74689
NODE_199_9	28	hypothetical protein	99.442	3	-0.30517
NODE_199_9	36	hypothetical protein	80.229	4	-0.74882
NODE_199_9	68	hypothetical protein	64.841	2	-0.16697
NODE_199_9	96	hypothetical protein	65.842	4	-0.83732
NODE_1_15	147	hypothetical protein	58.699	4	-0.38642
NODE_201_18	421	IDP1-isocitrate dehydrogenase (NADP+), mitochondrial	63.216	3	0.86122
NODE_201_18	181	IDP1-isocitrate dehydrogenase (NADP+), mitochondrial	73.632	2	-0.8757
NODE_201_18	165	IDP1-isocitrate dehydrogenase (NADP+), mitochondrial	62.799	3	-0.14611
NODE_201_6	368	40 kDa peptidyl-prolyl cis-trans isomerase	69.825	3	1.5837
NODE_201_6	351	40 kDa peptidyl-prolyl cis-trans isomerase	43.897	3	-0.28521
NODE_201_6	378	40 kDa peptidyl-prolyl cis-trans isomerase	65.224	2	-3.6429
NODE_209_13	159	hypothetical protein	47.894	2	-0.8851
NODE_209_13	239	hypothetical protein	63.062	3	0.25606
NODE_210_3	345	hypothetical protein	49.448	3	-1.8255
NODE_210_3	496	hypothetical protein	44.998	5	0.61212
NODE_211_10	238	serine/threonine-protein phosphatase PP1	51.135	3	-2.8657
NODE_211_10	141	serine/threonine-protein phosphatase PP1	50.978	2	-0.014827
NODE_211_12	98	hypothetical protein	60.735	3	-0.82166
NODE_211_12	39	hypothetical protein	89.356	3	0.38183
NODE_211_3	319	predicted protein	61.679	3	-0.62754
NODE_211_6	779	eukaryotic translation initiation factor 3 subunit EifCa, putative	80.688	3	-0.29194
NODE_212_7	7	hypothetical protein	57.175	3	-1.1633
NODE_214_12	38	hypothetical protein	62.088	2	0.69322
NODE_214_12	130	hypothetical protein	51.03	3	-0.18031
NODE_214_12	172	hypothetical protein	52.19	3	-0.24476
NODE_214_12	56	hypothetical protein	45.829	2	0.95279
NODE_214_16	104	eukaryotic ribosomal protein L18	120.15	4	-0.3931
NODE_214_16	96	eukaryotic ribosomal protein L18	55.776	4	2.4654
NODE_214_16	113	eukaryotic ribosomal protein L18	48.822	4	-0.062494
NODE_214_20	225	fimbrin	42.599	3	-1.865
NODE_214_5	773	hypothetical protein	46.88	3	-2.4801
NODE_214_8	2515	translational activator GCN1	53.453	3	-0.61002
NODE_216_20	247	uncharacterized protein	96.673	3	-0.8639
NODE_219_24	764	hypothetical protein	119.07	3	0.52107
NODE_219_24	841	hypothetical protein	99.283	2	0.18094
NODE_221_15	451	hypothetical protein	61.213	2	-1.4503
NODE_221_36	444	hypothetical protein	44.612	4	-0.52996
NODE_221_36	581	hypothetical protein	63.076	3	-1.9406
NODE_223_28	449	hypothetical protein	58.083	2	0.39245
NODE_223_28	435	hypothetical protein	105.2	2	0.44527
NODE_224_4	197	adenine nucleotide translocator	64.73	2	-0.64982
NODE_224_4	200	adenine nucleotide translocator	161.28	2	-0.050808
NODE_224_4	302	adenine nucleotide translocator	75.197	2	-0.37713
NODE_224_4	314	adenine nucleotide translocator	99.815	2	0.22073
NODE_224_4	111	adenine nucleotide translocator	83.862	2	-0.4847
NODE_224_4	300	adenine nucleotide translocator	69.721	3	0.70237
NODE_224_6	31	probable ribosomal protein	61.409	3	-1.5574
NODE_224_7	200	40S ribosomal protein S5	44.543	2	-2.0901

NODE_224_7	72	40S ribosomal protein S5	44.639	2	-1.8189
NODE_224_7	30	40S ribosomal protein S5	79.659	2	-0.53994
NODE_225_18	195	HRI1 domain protein	47.302	3	2.9818
NODE_225_25	274	putative six-bladed beta-propeller -like protein	52.247	2	0.49474
NODE_225_25	488	putative six-bladed beta-propeller -like protein	55.04	2	0.14616
NODE_228_10	257	predicted protein	70.438	3	1.3945
NODE_228_16	327	hypothetical protein	63.998	3	-1.0608
NODE_228_16	302	hypothetical protein	56.205	3	-1.8654
NODE_228_16	51	hypothetical protein	40.137	3	0.45779
NODE_228_18	378	vacuolar ATP synthase subunit B	41.482	3	-3.6459
NODE_228_4	530	hypothetical protein	42.314	3	-1.5095
NODE_229_36	136	proteosome beta subunit protease	103.08	2	-0.5078
NODE_2303_101	106	hypothetical protein	40.724	3	-0.21765
NODE_2303_18	629	probable translation elongation factor eEF-3	84.479	3	0.48868
NODE_2303_18	637	probable translation elongation factor eEF-3	82.287	3	0.60185
NODE_2303_18	346	probable translation elongation factor eEF-3	53.756	2	-0.080631
NODE_2303_18	471	probable translation elongation factor eEF-3	50.827	3	-1.1632
NODE_2303_27	253	hypothetical protein	70.783	3	3.2064
NODE_2303_28	90	glutaminyl-tRNA synthetase	48.468	3	3.5615
NODE_2303_28	260	glutaminyl-tRNA synthetase	101.65	3	0.45398
NODE_2303_34	212	dihydrolipoamide acetyltransferase component	71.451	3	-0.37615
NODE_2303_34	354	dihydrolipoamide acetyltransferase component	78.113	3	-1.0172
NODE_2303_34	196	dihydrolipoamide acetyltransferase component	67.08	3	-0.30793
NODE_2303_34	297	dihydrolipoamide acetyltransferase component	96.034	2	-1.3628
NODE_2303_78	399	hypothetical protein	58.172	2	0.43519
NODE_2307_15	88	succinate dehydrogenase iron-sulfur protein	46.069	2	-1.4653
NODE_230_11	58	acyl-CoA dehydrogenase	50.95	3	4.2703
NODE_230_16	8	uncharacterized protein	53.756	2	-1.1347
NODE_230_16	180	uncharacterized protein	90.827	3	2.5419
NODE_230_16	82	uncharacterized protein	75.819	2	1.809
NODE_230_18	209	SEC23/Sec24 family protein	55.437	3	4.0571
NODE_230_18	740	SEC23/Sec24 family protein	52.527	3	-0.14341
NODE_230_2	103	predicted protein	60.157	3	-0.25889
NODE_230_2	64	predicted protein	62.466	2	-1.446
NODE_230_2	236	predicted protein	56.548	2	-0.65068
NODE_230_5	393	vacuolar protease A	71.501	3	1.0813
NODE_230_5	320	vacuolar protease A	47.037	3	0.31554
NODE_230_9	350	Flap endonuclease 1	40.496	2	2.2378
NODE_232_2	149	40S ribosomal protein S6	65.179	4	-1.1178
NODE_232_2	131	40S ribosomal protein S6	84.653	4	-0.54595
NODE_232_2	217	40S ribosomal protein S6	67.646	3	1.9387
NODE_232_2	23	40S ribosomal protein S6	73.499	2	-0.5627
NODE_233_32	248	Nitrile-specifier protein 5	45.099	4	2.2803
NODE_234_15	17	importin alpha subunit	46.985	4	-0.94118
NODE_234_21	371	hypothetical protein	50.354	3	-1.4871
NODE_234_29	390	hypothetical protein	51.567	3	0.16455
NODE_234_29	202	hypothetical protein	51.066	3	0.56279
NODE_234_42	216	putative translation initiation factor protein	54.066	4	0.025514
NODE_238_17	462	methionyl-tRNA synthetase	40.432	3	0.048173
NODE_238_3	176	predicted protein	44.598	2	-0.39368
NODE_238_34	21	40S ribosomal protein S18	47.334	2	0.64268
NODE_238_34	111	40S ribosomal protein S18	97.965	2	-0.24733
NODE_238_34	20	40S ribosomal protein S18	44.377	3	-0.82241
NODE_238_35	34	UPF0023 family protein	52.579	2	0.79102
NODE_238_4	271	F-type H+-transporting ATPase subunit gamma	67.095	3	-1.9741
NODE_238_4	96	F-type H+-transporting ATPase subunit gamma	81.239	2	-0.94668

NODE_238_8	95	Calcium/calmodulin dependent protein kinase	58.172	2	-1.0215
NODE_238_8	117	Calcium/calmodulin dependent protein kinase	69.805	5	3.8148
NODE_23_10	254	dihydrolipoyl dehydrogenase	49.298	4	-0.86213
NODE_23_10	306	dihydrolipoyl dehydrogenase	95.264	2	-1.3489
NODE_23_10	20	dihydrolipoyl dehydrogenase	87.476	3	-0.55791
NODE_241_193	147	L-xylulose reductase	67.981	2	-1.457
NODE_241_193	69	L-xylulose reductase	66.989	2	-4.0865
NODE_241_193	18	L-xylulose reductase	73.26	2	-0.41789
NODE_241_193	186	L-xylulose reductase	60.55	3	-1.5704
NODE_241_193	235	L-xylulose reductase	51.762	2	0.20561
NODE_241_193	140	L-xylulose reductase	66.826	3	-0.34566
NODE_241_4	189	hypothetical protein	48.568	3	0.56176
NODE_241_4	169	hypothetical protein	48.711	3	-1.7321
NODE_241_81	212	hypothetical protein	71.501	3	0.95836
NODE_241_89	327	oligopeptide transporter	41.242	3	1.1552
NODE_241_89	404	oligopeptide transporter	80.706	2	0.30946
NODE_241_89	223	oligopeptide transporter	88.282	3	1.6024
NODE_241_91	52	putative catalase protein	93.374	3	-0.62749
NODE_241_91	330	putative catalase protein	64.103	3	-0.40733
NODE_243_2	394	Isotrichodermin C-15 hydroxylase	46.065	4	2.4688
NODE_246_37	347	putative sphingosine-1-phosphate lyase protein	75.316	3	-1.1487
NODE_246_40	122	Probable proteasome subunit alpha type 2	78.964	2	-0.95548
NODE_246_41	1360	Dicer-like protein 2	63.816	2	-0.45843
NODE_246_45	27	predicted protein	41.876	3	0.65925
NODE_246_46	455	conserved hypothetical protein	48.899	2	3.7854
NODE_246_50	370	predicted protein	48.513	2	0.45198
NODE_246_50	432	predicted protein	47.302	3	-2.8837
NODE_246_50	399	predicted protein	106.26	3	-2.4312
NODE_246_50	406	predicted protein	80.312	2	-0.80954
NODE_246_50	377	predicted protein	46.494	3	-1.1568
NODE_249_15	41	alkaline serine protease[Clonostachys rosea]	61.409	3	-2.5178
NODE_249_23	59	hypothetical protein	50.484	3	0.28562
NODE_250_22	424	hypothetical protein	50.805	2	0.0050258
NODE_251_6	379	hypothetical protein	43.672	3	0.35316
NODE_253_19	402	bifunctional purine biosynthesis protein	61.815	2	-1.2779
NODE_253_19	283	bifunctional purine biosynthesis protein	42.068	3	-0.099873
NODE_253_19	462	bifunctional purine biosynthesis protein	67.997	2	-1.689
NODE_253_30	1075	vacuolar sorting protein	69.188	4	0.122
NODE_253_30	587	vacuolar sorting protein	59.067	2	-2.1537
NODE_255_34	654	trifunctional tryptophan biosynthesis enzyme	56.205	3	0.22737
NODE_256_11	174	translation Initiation Factor Eif4e	46.156	3	0.2353
NODE_256_11	100	translation Initiation Factor Eif4e	51.445	3	-0.92877
NODE_256_2	43	histone acetyltransferase GCN5	56.514	3	-0.28171
NODE_2602_59	894	hydantoinase/oxoprolinase	42.629	3	3.5388
NODE_2615_18	468	Actin-related protein, ARP4 class	55.097	3	0.9292
NODE_2615_38	404	hypothetical protein	100.88	2	0.50149
NODE_2615_38	430	hypothetical protein	64.191	2	0.26269
NODE_2615_4	541	UTP-glucose-1-phosphate uridylyltransferase	85.294	3	0.23836
NODE_2615_4	343	UTP-glucose-1-phosphate uridylyltransferase	54.982	2	-0.021743
NODE_2615_48	138	hypothetical protein	40.278	3	3.3329
NODE_2615_55	370	predicted protein	42.336	3	-1.1245
NODE_2615_58	168	hypothetical protein	51.445	3	-0.86807
NODE_2615_62	9	predicted protein	52.579	3	-0.3031
NODE_2615_73	553	hypothetical protein	48.284	3	-0.51946
NODE_2615_73	512	hypothetical protein	128.88	2	-0.31325
NODE_2615_73	443	hypothetical protein	68.657	2	0.043441

NODE_2615_73	268	hypothetical protein	58.172	2	-1.0498
NODE_2615_73	92	hypothetical protein	67.113	2	-1.4072
NODE_2615_73	77	hypothetical protein	55.724	3	0.39223
NODE_2615_73	375	hypothetical protein	53.751	3	-0.466
NODE_2615_73	106	hypothetical protein	50.353	2	-0.15188
NODE_2615_73	105	hypothetical protein	57.047	3	-0.66268
NODE_2615_8	187	peptidase M16 family protein	40.242	3	4.1489
NODE_2615_86	145	hypothetical protein	60.019	3	-0.49026
NODE_2615_86	173	hypothetical protein	41.242	2	-1.809
NODE_2615_86	322	hypothetical protein	42.976	2	4.4175
NODE_2615_86	297	hypothetical protein	94.767	2	0.1587
NODE_2623_1	530	uncharacterized protein	92.039	2	0.018839
NODE_263_12	216	predicted protein	48.568	3	0.32619
NODE_263_24	207	diphosphomevalonate decarboxylase	53.998	3	-0.76721
NODE_268_24	218	condensin complex component SMC2	44.132	4	4.2738
NODE_269_23	348	sterol 24-C-methyltransferase	40.968	3	-1.5544
NODE_269_23	28	sterol 24-C-methyltransferase	107.56	3	-0.16909
NODE_269_24	253	hypothetical protein	54.259	3	-1.4582
NODE_269_30	20	probable NADH-ubiquinone oxidoreductase 21 kDa subunit	57.414	5	3.4592
NODE_270_10	204	predicted protein	55.064	3	0.16197
NODE_270_10	210	predicted protein	70.089	4	-0.45939
NODE_270_10	298	predicted protein	49.343	3	0.42628
NODE_270_10	46	predicted protein	89.171	2	-1.103
NODE_270_10	184	predicted protein	80.69	3	1.0472
NODE_270_11	530	asparagine synthetase	91.845	2	-0.62839
NODE_270_11	518	asparagine synthetase	90.614	2	0.84146
NODE_270_5	211	mitochondrial precursor protein import receptor tom70	44.318	3	0.78493
NODE_270_6	129	hypothetical protein	65.179	2	-1.3882
NODE_270_6	146	hypothetical protein	52.576	2	-1.4506
NODE_271_14	158	predicted protein	51.03	3	3.3463
NODE_271_14	57	predicted protein	103.13	3	2.7387
NODE_271_14	66	predicted protein	58.123	3	2.7387
NODE_271_14	260	predicted protein	69.92	3	-1.172
NODE_271_14	223	predicted protein	51.286	2	0.14615
NODE_273_10	33	hypothetical protein	62.287	3	0.57079
NODE_273_21	187	adenine phosphoribosyltransferase	80.688	3	-0.22344
NODE_275_11	425	uncharacterized protein	44.945	3	-0.35308
NODE_275_14	626	Non-robosomal peptide synthetase	46.159	3	-0.31795
NODE_275_6	186	ADP-ribosylation factor 6	49.715	2	0.25905
NODE_275_8	116	actin-like protein 2	47.712	2	-0.15591
NODE_276_24	155	general negative regulator of transcription subunit 4	42.21	3	-1.7724
NODE_276_30	1074	predicted protein	42.075	3	-0.36056
NODE_276_4	51	hypothetical protein	63.694	2	-1.6897
NODE_276_56	898	hypothetical protein	51.346	3	-0.0097664
NODE_278_2	432	glutamate decarboxylase	100.04	3	3.6378
NODE_278_2	414	glutamate decarboxylase	50.04	4	2.9106
NODE_279_10	90	predicted protein	60.598	3	0.52868
NODE_279_10	211	predicted protein	66.758	2	-3.3923
NODE_279_2	352	translation initiation factor 3subunit B	67.997	2	0.8787
NODE_279_2	419	translation initiation factor 3 subunit B	47.712	3	-0.75367
NODE_279_6	289	hypothetical protein	82.417	3	-1.048
NODE_279_6	310	hypothetical protein	72.096	2	-1.5361
NODE_279_6	78	hypothetical protein	74.611	3	0.0066968
NODE_280_43	2089	hypothetical protein	78.098	3	0.27294
NODE_280_45	633	Calpain-9	66.267	3	-0.59824

NODE_280_45	291	Calpain-9	63.216	3	-0.13172
NODE_280_45	529	Calpain-9	78.264	3	-0.0053774
NODE_280_45	600	Calpain-9	69.331	3	-0.7498
NODE_280_45	537	Calpain-9	51.726	3	-1.2593
NODE_2812_20	130	hypothetical protein	43.794	4	2.1016
NODE_2812_69	171	hypothetical protein	42.958	3	-0.24481
NODE_281_18	145	hypothetical protein	46.88	2	-1.8423
NODE_281_18	179	hypothetical protein	88.768	3	1.2685
NODE_288_24	1283	uncharacterized protein	41.876	3	0.083746
NODE_288_28	38	glycoside hydrolase family 31	52.591	3	-0.12967
NODE_288_44	460	hypothetical protein	63.691	3	3.7151
NODE_289_10	101	Ribosomal protein L27e	52.527	2	-0.39728
NODE_289_13	328	predicted protein	74.147	4	-1.789
NODE_289_13	179	predicted protein	68.557	3	-1.5683
NODE_289_13	230	predicted protein	96.745	3	2.7252
NODE_289_13	224	predicted protein	47.894	2	0.72072
NODE_289_13	236	predicted protein	45.614	4	-0.065626
NODE_289_13	175	predicted protein	69.721	3	1.3259
NODE_289_13	331	predicted protein	59.908	2	-1.6101
NODE_293_12	351	putative 3-ketoacyl- thiolase protein	77.662	3	0.25755
NODE_293_4	234	putative carbonic anhydrase protein	53.038	3	-1.6196
NODE_293_4	183	putative carbonic anhydrase protein	45.384	5	-1.6053
NODE_2951_8	25	histone 2A variant	67.334	2	-0.06877
NODE_2951_8	7	histone 2A variant	98.543	4	0.32809
NODE_2951_8	26	histone 2A variant	95.523	2	0.25783
NODE_2951_8	12	histone 2A variant	122.64	3	1.1311
NODE_2951_8	17	histone 2A variant	122.64	3	2.6401
NODE_2951_8	5	histone 2A variant	86.803	3	-0.78215
NODE_296_8	259	aldehyde dehydrogenase	130.98	3	-1.4261
NODE_296_8	134	aldehyde dehydrogenase	46.352	3	-1.348
NODE_297_4	362	uncharacterized protein	47.574	3	-1.0252
NODE_297_4	357	uncharacterized protein	47.574	3	-1.0252
NODE_29_13	68	predicted protein	42.336	2	-1.6956
NODE_29_25	445	predicted protein	46.352	3	-3.4358
NODE_29_25	1092	predicted protein	52.579	2	-1.728
NODE_29_25	964	predicted protein	43.761	3	-0.95884
NODE_29_30	186	hypothetical protein	58.172	3	-0.21945
NODE_29_30	129	hypothetical protein	56.404	2	-1.6717
NODE_29_31	21	hypothetical protein	52.185	3	-0.67729
NODE_29_36	251	F-actin capping protein beta subunit	48.568	3	0.64176
NODE_29_8	83	Copper amine oxidase 1	41.242	3	1.694
NODE_2_101	219	TCP-1/cpn60 chaperonin family protein	65.224	3	-0.39596
NODE_2_101	279	TCP-1/cpn60 chaperonin family protein	49.448	3	0.35137
NODE_2_43	143	hypothetical protein	63.816	3	-0.013701
NODE_2_65	1022	dihydrolipoamide succinyltransferase	83.948	2	-2.1038
NODE_2_71	464	transcriptional activator spt7	69.92	2	-1.8153
NODE_2_71	472	transcriptional activator spt7	69.92	2	-0.5103
NODE_2_71	212	transcriptional activator spt7	45.873	3	-1.0099
NODE_2_71	468	transcriptional activator spt7	69.92	2	-0.5103
NODE_2_86	36	hypothetical protein	79.986	3	0.72553
NODE_2_86	140	hypothetical protein	40.496	3	-0.43134
NODE_2_94	170	hypothetical protein	82.287	2	-1.6604
NODE_2_94	310	hypothetical protein	61.161	2	-0.48256
NODE_2_97	166	CBS domain protein	76.378	3	0.8746
NODE_300_5	535	hypothetical protein	62.466	3	0.28229
NODE_300_5	336	hypothetical protein	109.11	3	0.021949

NODE_300_9	262	sarcosine oxidase	71.877	3	-2.7498
NODE_301_68	137	uncharacterized protein	68.676	4	-1.7146
NODE_301_68	104	uncharacterized protein	43.897	2	0.84023
NODE_301_68	69	uncharacterized protein	47.894	3	-1.7297
NODE_301_68	100	uncharacterized protein	56.087	3	-1.767
NODE_302_5	458	peroxisomal carrier	57.532	3	2.3082
NODE_306_10	294	hypothetical protein	55.972	3	0.16604
NODE_307_15	49	Ribosomal protein L36e	56.514	2	-1.3618
NODE_307_18	276	putative 14-3-3 protein	59.198	3	0.22672
NODE_307_18	219	putative 14-3-3 protein	60.398	3	0.10894
NODE_307_18	289	putative 14-3-3 protein	88.394	4	-0.56819
NODE_307_18	213	putative 14-3-3 protein	72.29	2	-0.47863
NODE_307_18	186	putative 14-3-3 protein	54.898	3	4.4519
NODE_307_18	224	putative 14-3-3 protein	78.342	3	-0.51131
NODE_307_23	164	cytochrome c peroxidase precursor	70.056	3	1.2234
NODE_307_27	315	hypothetical protein	53.453	3	3.8921
NODE_307_27	99	hypothetical protein	40.278	3	-0.7659
NODE_307_27	395	hypothetical protein	47.774	3	-1.0043
NODE_308_22	54	hypothetical protein	70.399	3	-0.11121
NODE_30_137	375	Ketol-acid reductoisomerase, mitochondrial	63.181	2	0.27935
NODE_30_137	186	Ketol-acid reductoisomerase, mitochondrial	43.03	3	-1.8645
NODE_30_3	869	predicted protein	40.493	3	-1.7259
NODE_30_35	58	hypothetical protein FOXB_02295	49.85	3	0.91591
NODE_30_5	363	hypothetical protein	50.358	3	-1.621
NODE_30_5	323	hypothetical protein	69.338	2	-1.7888
NODE_30_5	111	hypothetical protein	46.001	2	-0.55345
NODE_30_70	99	hypothetical protein	53.625	4	3.4035
NODE_30_74	476	hypothetical protein	44.734	2	-0.79893
NODE_30_74	473	hypothetical protein	44.734	2	-0.79893
NODE_310_10	32	uncharacterized protein	84.498	3	-1.2635
NODE_311_12	191	profilin	79.659	2	-0.2832
NODE_312_19	395	hypothetical protein	63.216	2	1.5527
NODE_319_11	367	Serine/threonine-protein phosphatase T	67.997	2	-0.55256
NODE_319_5	221	elongation factor 1-gamma 1	61.657	3	-0.84803
NODE_319_5	412	elongation factor 1-gamma 1	64.711	2	-1.703
NODE_319_5	259	elongation factor 1-gamma 1	46.892	3	-0.40775
NODE_319_5	417	elongation factor 1-gamma 1	53.968	3	0.29416
NODE_319_5	269	elongation factor 1-gamma 1	42.059	4	-2.2719
NODE_319_5	231	elongation factor 1-gamma 1	61.344	3	0.22915
NODE_319_5	98	elongation factor 1-gamma 1	46.796	2	-1.1687
NODE_319_5	278	elongation factor 1-gamma 1	100.88	3	-1.599
NODE_319_5	96	elongation factor 1-gamma 1	53.493	3	-0.088327
NODE_31_20	338	hypothetical protein	73.918	3	-1.2303
NODE_320_47	611	alkaline phosphatase D	66.022	3	4.1228
NODE_320_52	441	hypothetical protein	48.284	2	3.1179
NODE_320_65	47	hypothetical protein	53.756	2	-0.46274
NODE_323_19	18	ATP-dependent RNA helicase	45.081	3	-0.80186
NODE_324_13	66	predicted protein	48.568	3	-0.4758
NODE_324_9	119	ATP synthase beta	71.148	3	-1.1635
NODE_324_9	152	ATP synthase beta	85.973	3	-0.1425
NODE_325_20	451	PLP-dependent enzyme involved in cell wall biogenesis	53.567	2	-1.9735
NODE_325_3	191	phosphoglycerate kinase	83.948	3	-0.91658
NODE_325_3	272	phosphoglycerate kinase	118.28	2	0.097396
NODE_325_3	49	phosphoglycerate kinase	51.03	3	1.2541
NODE_325_3	144	phosphoglycerate kinase	45.433	2	0.21001
NODE_325_3	192	phosphoglycerate kinase	96.342	3	0.96589

NODE_325_3	325	phosphoglycerate kinase	80.69	2	0.090243
NODE_325_3	15	phosphoglycerate kinase	98.105	2	-0.49033
NODE_326_13	275	hypothetical protein	72.705	3	-0.94601
NODE_326_18	183	hypothetical protein	64.52	4	-0.29567
NODE_326_4	753	hypothetical protein	70.908	2	-0.17968
NODE_328_12	181	hypothetical protein	53.756	2	-1.2996
NODE_328_12	177	hypothetical protein	75.819	2	-0.31674
NODE_328_12	91	hypothetical protein	43.594	3	0.96742
NODE_328_12	78	hypothetical protein	79.837	4	-1.5365
NODE_328_9	53	scf complex subunit skp1	81.017	3	1.6886
NODE_328_9	134	scf complex subunit skp1	41.513	3	3.1184
NODE_328_9	125	scf complex subunit skp1	59.067	2	-0.63618
NODE_329_8	191	glucose dehydrogenase, putative	53.453	4	-1.2709
NODE_329_8	219	glucose dehydrogenase, putative	45.68	3	2.8821
NODE_329_8	493	glucose dehydrogenase, putative	62.759	4	-0.59161
NODE_329_8	262	glucose dehydrogenase, putative	75.229	3	1.052
NODE_32_16	68	Fungal specific transcription factor domain containing protein	71.614	3	-0.14843
NODE_32_3	112	hypothetical protein	55.064	2	-1.6462
NODE_32_32	86	hypothetical protein	55.064	2	2.2974
NODE_32_32	91	hypothetical protein	42.599	3	1.0575
NODE_32_35	72	50S ribosomal subunit protein L15	104.94	3	-2.3395
NODE_330_19	365	bleomycin hydrolase	95.502	2	-1.8263
NODE_330_30	291	hydrolase	68.536	2	-0.87893
NODE_331_22	269	hypothetical protein	74.841	3	-1.2665
NODE_331_22	21	hypothetical protein	83.182	2	-0.12418
NODE_331_22	13	hypothetical protein	64.103	4	-2.0133
NODE_331_22	19	hypothetical protein	76.679	3	0.86877
NODE_331_23	109	predicted protein	61.409	3	-0.78799
NODE_331_32	205	hypothetical protein	51.286	3	-1.0325
NODE_331_36	588	hypothetical protein	78.324	2	0.012965
NODE_331_36	219	hypothetical protein	72.928	2	0.59896
NODE_331_36	593	hypothetical protein	41.242	2	0.60912
NODE_331_36	523	hypothetical protein	102.52	3	-0.55376
NODE_331_36	267	hypothetical protein	68.536	2	-4.4859
NODE_331_36	603	hypothetical protein	42.031	2	-0.77545
NODE_331_36	270	hypothetical protein	43.897	3	0.24935
NODE_332_11	431	Adaptor protein complex	84.817	2	0.3307
NODE_332_26	85	translation elongation factor EF-1	67.646	3	-0.029706
NODE_332_26	394	translation elongation factor EF-1	44.543	3	-0.39537
NODE_332_26	377	translation elongation factor EF-1	85.813	3	0.073358
NODE_332_26	407	translation elongation factor EF-1	42.118	3	-1.9081
NODE_332_26	438	translation elongation factor EF-1	44.252	2	-0.13096
NODE_332_26	254	translation elongation factor EF-1	96.143	3	0.25371
NODE_332_26	178	translation elongation factor EF-1	83.948	2	-1.0145
NODE_332_26	37	translation elongation factor EF-1	74.141	3	0.52667
NODE_332_42	556	AreA-AC	54.784	2	-0.49237
NODE_332_46	545	predicted protein	52.576	3	-1.9164
NODE_332_66	22	hypothetical protein	97.203	3	-0.77376
NODE_332_66	10	hypothetical protein	59.252	4	-0.70846
NODE_335_19	59	40S ribosomal protein S17	80.706	3	0.13668
NODE_335_19	32	40S ribosomal protein S17	70.808	2	-0.68881
NODE_335_19	49	40S ribosomal protein S17	74.611	3	-0.71304
NODE_335_19	19	40S ribosomal protein S17	87.639	2	0.19299
NODE_335_19	14	40S ribosomal protein S17	72.2	2	-0.035944
NODE_33_11	172	proteinase T-like protein	52.49	3	-1.9287
NODE_33_11	339	proteinase T-like protein	55.453	3	-0.55284

NODE_33_11	179	proteinase T-like protein	58.172	3	-0.42721
NODE_340_3	652	predicted protein	41.911	4	1.842
NODE_340_3	286	predicted protein	43.69	4	-0.3891
NODE_340_3	871	predicted protein	75.764	5	3.9702
NODE_340_3	101	predicted protein	80.102	2	1.3789
NODE_340_3	798	predicted protein	82.417	2	-0.024685
NODE_340_3	29	predicted protein	85.469	3	0.86708
NODE_340_3	279	predicted protein	67.997	3	-2.1058
NODE_340_62	198	conserved hypothetical protein	48.376	3	-1.4755
NODE_341_4	699	hypothetical protein	87.138	3	-0.86446
NODE_345_10	252	hypothetical protein CHGG_01487	77.185	2	-0.39022
NODE_345_7	278	hypothetical protein	61.213	2	0.30471
NODE_345_7	290	hypothetical protein	81.972	2	0.28035
NODE_349_36	296	RNA recognition domain-containing protein	76.345	2	-0.19523
NODE_349_42	240	isocitrate dehydrogenase [NAD] subunit 1, mitochondrial	72.2	3	-0.15213
NODE_349_42	296	isocitrate dehydrogenase [NAD] subunit 1, mitochondrial	92.051	3	1.2909
NODE_349_42	302	isocitrate dehydrogenase [NAD] subunit 1, mitochondrial	84.507	3	-0.47836
NODE_349_43	131	lysyl-tRNA synthetase	47.844	3	-0.76475
NODE_351_5	572	uncharacterized protein	49.448	3	1.517
NODE_351_5	627	uncharacterized protein	69.03	2	0.79071
NODE_351_5	583	uncharacterized protein	69.721	3	-1.5579
NODE_351_5	648	uncharacterized protein	85.554	2	-0.86588
NODE_353_12	252	ribosomal protein L6	57.175	2	-1.3851
NODE_353_12	125	ribosomal protein L6	44.511	3	-1.46
NODE_353_12	199	ribosomal protein L6	68.786	2	1.5883
NODE_353_12	126	ribosomal protein L6	59.116	4	0.47239
NODE_353_8	536	probable phosphoacetylglucosamine mutase	45.368	2	-0.42039
NODE_353_8	529	probable phosphoacetylglucosamine mutase	72.898	3	3.7758
NODE_359_23	102	SNF2 family protein	83.647	2	-0.16582
NODE_359_25	212	predicted protein	81.017	3	-0.048404
NODE_359_25	960	predicted protein	61.962	3	-0.53898
NODE_359_25	351	predicted protein	56.258	3	0.52301
NODE_359_27	102	hypothetical protein	58.848	3	-1.2588
NODE_359_5	8	60S ribosomal protein L5	50.354	2	0.46625
NODE_359_5	27	60S ribosomal protein L5	90.913	2	-0.16799
NODE_359_5	264	60S ribosomal protein L5	73.632	3	-1.4119
NODE_359_5	43	60S ribosomal protein L5	58.782	2	-3.1677
NODE_359_5	41	60S ribosomal protein L5	80.231	2	-0.35046
NODE_359_7	422	hypothetical protein	44.863	3	-0.10683
NODE_360_18	395	dihydroorotate oxidase	46.797	2	4.0574
NODE_360_29	847	ubiquitin-activating enzyme E1 family protein	74.987	2	1.5376
NODE_360_29	987	ubiquitin-activating enzyme E1 family protein	50.354	3	1.75
NODE_360_48	286	hypothetical protein	50.354	3	-0.39078
NODE_360_48	285	hypothetical protein	50.354	3	-0.39078
NODE_360_48	288	hypothetical protein	50.354	3	-0.39078
NODE_360_57	486	hypothetical protein	56.527	3	-2.4997
NODE_360_57	449	hypothetical protein	44.848	5	1.872
NODE_362_30	284	Nucleotide-binding, alpha-beta plait	91.961	3	-1.3982
NODE_362_4	94	hypothetical protein	68.657	2	1.5835
NODE_368_10	173	probable GTP-binding protein ypt5	76.228	2	0.25265
NODE_368_2	159	Ribosomal protein S17	42.843	3	0.2313
NODE_368_2	119	Ribosomal protein S17	93.345	2	-0.094545
NODE_368_2	73	Ribosomal protein S17	46.704	3	0.69886
NODE_368_2	196	Ribosomal protein S17	44.238	3	-0.73777

NODE_368_2	168	Ribosomal protein S17	54.309	4	0.31294
NODE_369_9	227	hypothetical protein	73.138	3	3.1401
NODE_370_2	677	hypothetical protein	46.318	3	-0.55077
NODE_372_2	58	RNA recognition domain-containing protein	45.614	4	-0.51941
NODE_374_8	252	hypothetical protein	48.284	2	-0.50743
NODE_374_8	820	hypothetical protein	85.554	3	1.6657
NODE_374_8	259	hypothetical protein	51.528	2	-0.96171
NODE_374_8	486	hypothetical protein	88.181	2	0.032607
NODE_374_8	245	hypothetical protein	54.276	5	-1.1805
NODE_375_6	426	hypothetical protein	57.347	3	-0.70322
NODE_375_6	290	hypothetical protein	79.201	5	-0.50891
NODE_375_8	140	flavocytochrome c	68.95	3	0.049712
NODE_378_29	584	NCP1-NADPH-cytochrome P450 reductase	72.2	2	-0.46838
NODE_378_84	137	predicted protein	54.259	3	-1.1728
NODE_378_89	215	hypothetical protein	49.298	3	3.8659
NODE_37_57	44	GNAT family acetyltransferase, putative	81.709	3	-0.12347
NODE_382_2	59	cold-shock protein	50.305	3	-2.5424
NODE_386_6	288	uncharacterized protein	42.317	2	-0.22719
NODE_38_18	57	GBB_CRYPA Guanine nucleotide-binding protein beta subunit	62.055	3	-3.1144
NODE_38_18	73	GBB_CRYPA Guanine nucleotide-binding protein beta subunit	64.244	4	-1.0368
NODE_38_18	29	GBB_CRYPA Guanine nucleotide-binding protein beta subunit	56.916	2	-0.87162
NODE_38_21	264	d-3-phosphoglyceratedehydrogenase	62.466	3	-0.25293
NODE_38_29	620	hypothetical protein	46.89	3	-1.0505
NODE_38_33	287	hypothetical protein	92.19	3	0.40138
NODE_38_33	96	hypothetical protein	44.614	2	-0.48447
NODE_38_36	100	hypothetical protein	58.885	3	3.9255
NODE_38_5	436	T-complex protein 1 subunit epsilon	40.767	3	-1.2669
NODE_38_5	195	T-complex protein 1 subunit epsilon	50.088	5	-3.2092
NODE_38_7	124	molecular chaperone bip1	79.474	3	-1.1194
NODE_38_7	159	molecular chaperone bip1	48.568	2	0.22491
NODE_38_7	278	molecular chaperone bip1	72.2	2	-0.40621
NODE_38_7	168	molecular chaperone bip1	64.82	2	-1.1732
NODE_38_7	358	molecular chaperone bip1	46.249	3	-0.55936
NODE_38_72	371	beta-tubulin	64.454	3	-2.0595
NODE_38_72	50	beta-tubulin	40.857	3	4.0389
NODE_38_72	316	beta-tubulin	48.513	2	-3.1608
NODE_393_3	163	casein kinase I, putative	66.023	5	0.16228
NODE_393_3	125	casein kinase I, putative	47.429	4	0.28751
NODE_396_7	899	hypothetical protein	72.848	2	-0.26955
NODE_396_7	939	hypothetical protein	64.52	3	-0.18993
NODE_396_7	946	hypothetical protein	64.52	3	-0.18993
NODE_396_9	238	60S ribosomal protein L8	49.3	2	0.011713
NODE_396_9	243	60S ribosomal protein L8	64.52	3	0.52675
NODE_396_9	255	60S ribosomal protein L8	71.176	2	-0.22001
NODE_396_9	122	60S ribosomal protein L8	70.089	3	-0.099234
NODE_396_9	223	60S ribosomal protein L8	77.894	2	-0.62518
NODE_396_9	222	60S ribosomal protein L8	42.599	3	-0.6147
NODE_398_13	179	uncharacterized protein	82.651	4	0.16776
NODE_400_8	789	predicted protein	50.108	3	-0.35517
NODE_403_36	260	hypothetical protein	40.015	3	0.24331
NODE_403_60	177	hypothetical protein	45.347	3	0.70688
NODE_404_15	195	predicted protein	44.511	3	1.119
NODE_405_31	279	norsolorinic acid reductase	51.066	2	1.2022
NODE_405_46	56	hypothetical protein	44.598	2	-0.63242

NODE_406_12	737	hypothetical protein	49.632	3	-0.2042
NODE_406_12	754	hypothetical protein	43.03	3	-0.91282
NODE_406_4	102	Triosephosphate isomerase	44.252	3	-0.38863
NODE_406_4	188	Triosephosphate isomerase	114.87	2	-1.9833
NODE_406_4	176	Triosephosphate isomerase	89.624	3	0.77282
NODE_407_22	300	Glycylpeptide N-tetradecanoyltransferase	52.247	3	1.0607
NODE_407_34	140	hypothetical protein	54.343	3	2.561
NODE_407_8	20	predicted protein	54.898	4	0.29364
NODE_408_16	475	hypothetical protein X797_001934	68.413	3	3.8776
NODE_408_34	112	hypothetical protein	48.568	3	-1.9192
NODE_408_34	49	hypothetical protein	115.34	3	-0.20078
NODE_408_34	117	hypothetical protein	63.32	3	-0.38094
NODE_412_8	74	Long-chain acyl-CoA synthetases & Acyl-protein synthetase	76.843	3	-0.96769
NODE_412_8	375	Long-chain acyl-CoA synthetases & Acyl-protein synthetase	54.898	2	-0.87249
NODE_415_1	264	hypothetical protein	78.964	3	-2.1424
NODE_415_1	129	hypothetical protein	48.568	4	-1.0823
NODE_415_2	35	hypothetical protein	60.255	2	-0.80608
NODE_415_2	38	hypothetical protein	50.897	2	1.4957
NODE_415_2	46	hypothetical protein	85.676	3	0.71296
NODE_415_2	24	hypothetical protein	90.906	3	-0.99868
NODE_415_8	427	putative aspartate aminotransferase	88.338	2	-0.080689
NODE_415_8	419	putative aspartate aminotransferase	50.46	3	0.38238
NODE_415_8	325	putative aspartate aminotransferase	49.988	2	0.04967
NODE_417_11	21	60S ribosomal protein L126	75.819	3	-1.912
NODE_417_11	77	60S ribosomal protein L126	66.19	2	-2.4516
NODE_417_11	89	60S ribosomal protein L126	59.975	4	-0.71511
NODE_417_11	37	60S ribosomal protein L126	71.176	3	0.38022
NODE_417_11	110	60S ribosomal protein L126	66.004	3	-0.28901
NODE_417_20	22	predicted protein	56.258	3	-0.85359
NODE_417_21	340	hypothetical protein	60.102	3	-0.40682
NODE_417_21	396	hypothetical protein	83.404	3	-0.86351
NODE_417_21	242	hypothetical protein	63.979	4	-1.3411
NODE_417_7	399	ribose-phosphate pyrophosphokinase	69.314	3	-0.044798
NODE_41_10	248	60S ribosomal protein L13	42.314	2	-1.0593
NODE_41_10	111	60S ribosomal protein L13	77.662	2	-0.57444
NODE_41_27	74	60S ribosomal protein L10	103.55	2	-1.3063
NODE_41_27	101	60S ribosomal protein L10	58.32	2	-1.4068
NODE_41_27	78	60S ribosomal protein L10	89.296	3	-0.5344
NODE_420_14	188	hypothetical protein MAA_03206	58.172	3	-0.071676
NODE_422_8	311	putative proline-specificpeptidase protein	86.114	3	-0.92023
NODE_42_46	211	nucleoside-diphosphate-sugar epimerase	70.942	3	-0.37156
NODE_433_25	408	hypothetical protein	58.246	3	-3.0475
NODE_433_25	362	hypothetical protein	50.897	3	3.1005
NODE_433_25	190	hypothetical protein	55.353	2	1.1263
NODE_433_25	723	hypothetical protein	101.35	3	-0.17517
NODE_433_25	424	hypothetical protein	45.342	3	-0.96281
NODE_433_25	449	hypothetical protein	85.813	2	-1.8578
NODE_434_14	336	GMC oxidoreductase	60.489	3	2.6751
NODE_438_20	95	ribosomal S3Ae family protein	40.086	4	-2.1531
NODE_438_20	56	ribosomal S3Ae family protein	66.004	2	-0.25552
NODE_438_20	153	ribosomal S3Ae family protein	50.222	2	2.3306
NODE_438_22	58	GTP cyclohydrolase II	79.69	3	3.4656
NODE_438_33	354	POT family protein	40.477	3	-1.6459
NODE_438_37	194	predicted protein	49.3	2	0.22251
NODE_438_4	33	killer toxin subunits alpha/beta	40.625	4	2.9468

NODE_439_101	33	ATP synthase D chain	111.79	3	-1.3943
NODE_439_101	63	ATP synthase D chain	58.079	3	2.021
NODE_439_101	73	ATP synthase D chain	86.772	2	-2.7883
NODE_439_110	59	related to signal recognition particle receptor alpha subunit	62.781	4	2.9527
NODE_439_115	92	uncharacterized protein	55.676	2	-1.6451
NODE_439_22	426	vacuolar serine protease	75.416	3	-0.94505
NODE_439_22	278	vacuolar serine protease	86.497	3	-1.0917
NODE_439_22	249	vacuolar serine protease	43.68	3	-0.54778
NODE_439_22	235	vacuolar serine protease	52.898	4	-3.0936
NODE_439_22	242	vacuolar serine protease	96.948	2	-0.40911
NODE_439_75	283	hypothetical protein	41.399	3	-2.7899
NODE_439_75	279	hypothetical protein	76.679	3	-1.1248
NODE_439_8	296	uncharacterized protein	56.514	3	0.46256
NODE_439_87	112	hypothetical protein	45.992	3	-2.4302
NODE_439_87	56	hypothetical protein	83.862	2	-0.23025
NODE_439_99	92	ribosomal protein L14	83.182	2	-0.098344
NODE_439_99	42	ribosomal protein L14	65.179	3	-1.5185
NODE_439_99	98	ribosomal protein L14	73.233	2	0.317
NODE_440_4	22	Ribosomal protein L21e	71.614	3	-0.63164
NODE_440_4	27	Ribosomal protein L21e	99.844	3	-2.4968
NODE_440_4	45	Ribosomal protein L21e	102.52	2	-0.72091
NODE_440_4	96	Ribosomal protein L21e	72.2	3	-0.81595
NODE_440_5	113	Ribosomal protein S4/S9	52.576	3	0.68992
NODE_440_9	618	predicted protein	64.82	2	0.11633
NODE_441_1	37	predicted protein	48.284	3	0.42323
NODE_441_10	123	histone H3	73.881	3	0.26204
NODE_441_10	28	histone H3	98.407	3	-1.4397
NODE_441_10	57	histone H3	184.03	3	2.2467
NODE_441_10	80	histone H3	51.286	2	0.31503
NODE_441_10	15	histone H3	115.7	2	1.1025
NODE_441_10	37	histone H3	91.789	3	1.7373
NODE_441_10	24	histone H3	127.93	2	0.39245
NODE_441_10	10	histone H3	105.46	3	-1.1772
NODE_441_10	19	histone H3	127.93	3	1.5382
NODE_441_11	6	hypothetical protein	134.61	2	-0.29721
NODE_441_11	92	hypothetical protein	129.37	2	-1.9089
NODE_441_11	32	hypothetical protein	76.728	2	-1.3961
NODE_441_11	9	hypothetical protein	134.61	2	-0.29721
NODE_441_11	13	hypothetical protein	136.99	3	-0.35486
NODE_441_11	78	hypothetical protein	136.7	2	-1.0366
NODE_441_11	17	hypothetical protein	136.99	3	-0.35486
NODE_441_27	598	predicted protein	46.838	3	2.4651
NODE_441_27	707	predicted protein	63.419	3	0.21683
NODE_441_27	418	predicted protein	57.973	3	-1.1678
NODE_441_27	416	predicted protein	66.19	3	-0.46272
NODE_441_39	507	hypothetical protein	71.176	3	2.1227
NODE_441_40	11	hypothetical protein	60.255	2	-1.1025
NODE_443_6	108	endoribonuclease L-PSP	78.342	2	-1.5557
NODE_444_7	7	uncharacterized protein	66.989	3	0.01328
NODE_446_4	83	Hsp90 associated co-chaperone	79.659	4	-0.10447
NODE_446_5	45	hypothetical protein	52.247	3	-2.0621
NODE_446_5	160	hypothetical protein	67.214	3	-2.0411
NODE_446_9	103	cobalamin-independent methionine synthase	61.444	3	4.1175
NODE_446_9	40	cobalamin-independent methionine synthase	80.632	2	0.24083
NODE_446_9	414	cobalamin-independent methionine synthase	60.019	2	-1.0548
NODE_446_9	20	cobalamin-independent methionine synthase	59.198	2	0.62743

NODE_446_9	407	cobalamin-independent methionine synthase	51.286	2	-1.524
NODE_449_6	227	Eukaryotic translation initiation factor3 subunit	99.283	2	-1.1748
NODE_44_31	512	hypothetical protein	52.49	3	1.531
NODE_44_55	333	predicted protein	64.52	3	-1.4142
NODE_44_55	493	predicted protein	46.88	3	1.3644
NODE_44_74	141	mitochondrial peroxiredoxin PRX1	59.908	2	-0.84384
NODE_455_18	95	40S ribosomal protein S14	71.176	3	1.243
NODE_455_26	644	predicted protein	61.09	3	1.6749
NODE_455_40	62	hypothetical protein	125.97	3	-2.787
NODE_455_40	48	hypothetical protein	67.897	3	-2.2941
NODE_455_40	47	hypothetical protein	126.19	2	-1.5105
NODE_463_27	308	D-isomer specific 2-hydroxyacid dehydrogenase	50.358	3	2.3722
NODE_464_3	115	CMGC/CK2 protein kinase	46.069	4	-1.2519
NODE_469_42	1294	multisynthetase complex auxiliary component p43	54.09	3	-0.007998
NODE_469_42	1277	multisynthetase complex auxiliary component p43	61.11	2	-0.061052
NODE_46_1	80	glycosyltransferase family 35	40.475	2	-1.041
NODE_46_6	399	hypothetical protein	61.657	2	4.4335
NODE_476_16	60	hypothetical protein	59.35	3	-2.8233
NODE_476_6	113	hypothetical protein	82.029	3	-0.33038
NODE_476_7	174	predicted protein	45.614	3	-0.45473
NODE_476_7	182	predicted protein	67.385	2	1.9703
NODE_486_16	48	predicted protein	51.726	3	4.2513
NODE_486_19	205	actin-binding protein	91.589	4	-0.59088
NODE_486_21	493	2,3-bisphosphoglycerate-independent phosphoglycerate mutase	88.427	3	-0.33982
NODE_486_21	852	2,3-bisphosphoglycerate-independent phosphoglycerate mutase	70.942	3	0.78157
NODE_487_23	71	60S ribosomal protein L27a	41.621	3	-0.097788
NODE_487_23	94	60S ribosomal protein L27a	70.889	3	0.76384
NODE_487_23	43	60S ribosomal protein L27a	80.455	3	-0.29534
NODE_488_11	57	hypothetical protein	52.247	2	-0.88957
NODE_489_13	303	stress-induced-phosphoprotein 1	55.261	3	-0.32418
NODE_489_13	264	stress-induced-phosphoprotein 1	46.462	2	1.2012
NODE_489_18	325	hypothetical protein	59.013	3	0.24489
NODE_489_25	578	predicted protein	92.38	4	-1.7749
NODE_489_25	194	predicted protein	53.124	2	-1.0912
NODE_489_4	212	predicted protein	66.246	3	-0.32998
NODE_489_4	221	predicted protein	56.404	3	0.30833
NODE_489_4	101	predicted protein	83.404	4	-0.12961
NODE_489_4	139	predicted protein	53.448	3	-2.4721
NODE_489_4	133	predicted protein	79.652	3	2.0736
NODE_48_27	216	expressed protein	40.352	3	-3.8024
NODE_48_76	298	hypothetical protein	47.712	3	-0.39325
NODE_48_82	221	4-aminobutyrate aminotransferase	45.873	4	0.92677
NODE_48_82	387	4-aminobutyrate aminotransferase	63.624	3	0.28511
NODE_48_82	216	4-aminobutyrate aminotransferase	54.023	2	-2.702
NODE_490_13	151	predicted protein	48.794	3	0.85874
NODE_490_19	126	hypothetical protein	84.173	3	-0.41333
NODE_490_22	18	ribosomal protein L38e	80.219	3	-0.72323
NODE_490_30	118	hypothetical protein	44.511	3	-0.28394
NODE_496_11	204	urease	90.15	3	0.091515
NODE_496_12	401	hypothetical protein	40.941	2	1.041
NODE_496_12	296	hypothetical protein	131.17	3	-2.8316
NODE_496_12	372	hypothetical protein	40.986	3	-0.40752
NODE_496_12	410	hypothetical protein	70.942	2	1.0453
NODE_496_9	112	hypothetical protein	109.72	4	-0.46985
NODE_496_9	8	hypothetical protein	80.316	3	-0.0077122

NODE_496_9	13	hypothetical protein	80.316	3	-0.0077122
NODE_496_9	12	hypothetical protein	80.316	3	-0.0077122
NODE_496_9	126	hypothetical protein	109.72	5	1.0564
NODE_498_10	133	hypothetical protein	41.796	3	1.2703
NODE_498_23	212	C2H2 finger domain protein (Gli3), putative	71.085	3	-1.0035
NODE_49_6	161	hypothetical protein	67.385	2	-1.9136
NODE_49_7	359	hypothetical protein	43.592	3	-1.2682
NODE_49_7	357	hypothetical protein	40.941	3	-0.69143
NODE_49_8	166	probable electron transfer flavoprotein alpha chain precursor	42.947	3	-0.25813
NODE_507_14	114	hypothetical protein	40.475	3	3.7018
NODE_510_43	257	hypothetical protein	53.448	3	-3.3614
NODE_510_5	244	hypothetical protein	71.08	3	-1.0133
NODE_510_5	56	hypothetical protein	44.863	2	0.0011818
NODE_510_5	318	hypothetical protein	46.069	3	-1.3128
NODE_510_5	171	hypothetical protein	94.767	2	-0.45028
NODE_510_5	5	hypothetical protein	88.819	3	1.465
NODE_514_3	367	saccharopine dehydrogenase	50.473	3	-0.55705
NODE_514_3	145	saccharopine dehydrogenase	44.318	3	1.3351
NODE_514_3	385	saccharopine dehydrogenase	51.76	3	-1.1552
NODE_514_3	303	saccharopine dehydrogenase	44.406	2	-2.2251
NODE_514_33	52	hypothetical protein	76.679	2	-1.4248
NODE_514_33	40	hypothetical protein	57.532	2	-0.18811
NODE_514_33	71	hypothetical protein	80.752	3	0.8666
NODE_514_33	24	hypothetical protein	46.069	3	-1.5016
NODE_514_33	102	hypothetical protein	77.192	3	0.28844
NODE_514_40	35	RNA binding protein	40.941	3	4.366
NODE_516_6	210	RSC complex subunit Rsc7	47.228	3	-0.26669
NODE_516_6	125	RSC complex subunit Rsc7	57.149	2	-0.649
NODE_516_6	160	RSC complex subunit Rsc7	93.096	2	-0.1323
NODE_51_21	455	eukaryotic translation initiation factor 3	95.417	3	0.73615
NODE_51_27	54	hypothetical protein	57.532	3	-1.0366
NODE_51_28	280	predicted protein	52.576	3	-0.80849
NODE_51_36	200	hypothetical protein	52.49	3	-0.20981
NODE_522_3	145	peptide chain release factor, subunit 1	41.448	3	-1.2722
NODE_522_3	138	peptide chain release factor,subunit 1	41.242	3	-0.422
NODE_522_36	348	predicted protein	84.297	3	-0.43219
NODE_522_36	24	predicted protein	40.187	3	3.2081
NODE_522_36	26	predicted protein	42.976	3	0.11536
NODE_522_36	293	predicted protein	60	2	-1.0445
NODE_522_5	145	60S ribosomal protein L2	56.81	2	-0.45332
NODE_524_3	553	hypothetical protein	59.067	4	0.81676
NODE_525_17	132	Pre-mRNA-processing protein prp40	55.261	3	3.4983
NODE_525_26	461	DNA replication licensing factor mcm7	40.941	3	1.5292
NODE_525_28	193	ribosome biogenesis protein	63.216	2	0.069561
NODE_52_12	50	hypothetical protein	62.582	4	0.10392
NODE_530_2	116	hypothetical protein	53.08	3	-1.7052
NODE_530_2	151	hypothetical protein	58.079	3	-0.30392
NODE_530_2	111	hypothetical protein	50.077	3	-3.5288
NODE_533_16	120	hypothetical protein	72.434	3	-1.9776
NODE_53_14	261	hypothetical protein	42.802	3	-1.2628
NODE_53_14	338	hypothetical protein	55.261	3	-0.47995
NODE_53_16	689	putative 3-isopropylmalatedehydratase protein	42.599	3	-0.51564
NODE_53_5	259	Small GTPase superfamily, Rho type	64.121	3	0.020613
NODE_53_5	248	Small GTPase superfamily, Rho type	46.249	3	0.2656
NODE_548_18	240	Phosducin family protein	92.838	2	-2.1924
NODE_54_11	370	electron transfer flavoprotein	74.611	3	0.40172

NODE_54_12	91	TATA-box-binding protein	77.062	3	-0.16248
NODE_54_8	152	4 family polyadenylate binding protein	47.68	3	3.5061
NODE_54_8	476	4 family polyadenylate binding protein	70.889	2	-2.9713
NODE_54_8	225	4 family polyadenylate binding protein	46.462	3	0.89253
NODE_54_8	126	4 family polyadenylate binding protein	59.227	3	-1.3417
NODE_54_8	143	4 family polyadenylate binding protein	72.29	2	0.16081
NODE_551_1	191	trehalose synthase	67.207	3	1.3657
NODE_551_1	602	trehalose synthase	48.899	5	0.28089
NODE_552_5	268	hypothetical protein	42.947	4	0.3432
NODE_556_4	262	hypothetical protein	63.473	3	-0.58514
NODE_558_12	630	hypothetical protein	45.28	2	2.117
NODE_558_17	85	Putative methionine aminopeptidase 1	79.659	3	0.70712
NODE_558_25	13	uncharacterized protein	50.04	3	-0.83659
NODE_559_14	147	hypothetical protein	70.028	2	0.11454
NODE_55_27	360	glutaryl-CoA dehydrogenase	51.495	4	-2.178
NODE_55_27	317	glutaryl-CoA dehydrogenase	42.843	3	-1.7096
NODE_561_22	183	hypothetical protein	56.569	3	-0.60571
NODE_561_22	144	hypothetical protein	51.436	3	-1.634
NODE_565_2	230	probable CBF1-centromere binding factor 1	44.318	2	0.070789
NODE_566_3	87	hypothetical protein	56.205	3	0.23015
NODE_566_3	65	hypothetical protein	56.514	3	2.7587
NODE_566_7	440	hypothetical protein	71.176	3	3.509
NODE_566_7	53	hypothetical protein	58.699	3	0.90104
NODE_567_11	90	cytochrome c	63.306	3	-2.1772
NODE_567_11	15	cytochrome c	53.028	2	1.3414
NODE_569_3	129	uncharacterized protein	56.139	3	0.047659
NODE_569_5	163	sulfate adenylyltransferase	82.287	3	1.3564
NODE_569_5	257	sulfate adenylyltransferase	80.014	4	0.39317
NODE_569_5	121	sulfate adenylyltransferase	41.257	4	-1.4785
NODE_56_11	156	hypothetical protein	42.629	3	-0.11087
NODE_57_29	207	Peroxidase/catalase	70.056	3	-1.149
NODE_58_26	130	Rab-GDP dissociation inhibitor	67.76	3	-0.033659
NODE_58_26	98	Rab-GDP dissociation inhibitor	73.499	3	-0.043532
NODE_58_26	275	Rab-GDP dissociation inhibitor	81.974	4	-1.2451
NODE_58_26	299	Rab-GDP dissociation inhibitor	80.361	4	3.7081
NODE_58_31	230	Cys(2)-His(2) zinc finger domain protein	85.676	4	-2.5211
NODE_58_41	40	putative phospholipase d protein	46.352	2	0.69647
NODE_58_51	137	heat shock 70 kDa protein	47.726	3	3.485
NODE_58_51	567	heat shock 70 kDa protein	55.084	3	1.3334
NODE_58_51	154	heat shock 70 kDa protein	43.592	3	-0.15469
NODE_58_51	489	heat shock 70 kDa protein	87.476	2	-2.7538
NODE_58_51	570	heat shock 70 kDa protein	46.844	2	-1.1035
NODE_58_51	285	heat shock 70 kDa protein	68.224	3	-0.96314
NODE_58_51	121	heat shock 70 kDa protein	55.353	2	-0.92369
NODE_595_2	568	arginyl-tRNA synthetase	42.976	4	-3.1788
NODE_595_3	465	hypothetical protein	110.8	3	-0.096008
NODE_598_1	295	3-beta hydroxysteroiddehydrogenase protein	46.352	3	0.049039
NODE_604_2	126	Cell division control protein 2 (Cyclin-dependent protein kinase)	51.276	2	-1.1955
NODE_604_21	588	alcohol oxidase	43.297	3	0.7689
NODE_604_21	272	alcohol oxidase	46.352	2	-0.8386
NODE_604_21	549	alcohol oxidase	97.635	2	-0.14381
NODE_604_21	517	alcohol oxidase	72.705	2	-1.1475
NODE_604_21	129	alcohol oxidase	71.379	3	-2.2177
NODE_604_21	211	alcohol oxidase	44.925	3	-0.78472
NODE_604_21	200	alcohol oxidase	79.639	3	-1.0791
NODE_604_21	66	alcohol oxidase	47.639	4	1.1185

NODE_604_21	214	alcohol oxidase	49.448	2	0.88087
NODE_606_19	185	COP9 signalosome complex subunit 12	60.91	3	-0.46075
NODE_606_20	108	probable 60S large subunit ribosomal protein	84.753	3	0.31668
NODE_606_20	113	probable 60S large subunit ribosomal protein	79.474	4	-0.6562
NODE_608_11	168	translation initiation factor eIF3 p40 subunit	60.255	3	-0.8172
NODE_608_12	191	adenosylhomocysteinase	82.261	3	-0.35324
NODE_608_12	39	adenosylhomocysteinase	76.868	3	0.32886
NODE_608_12	207	adenosylhomocysteinase	96.668	3	-0.97608
NODE_608_12	151	adenosylhomocysteinase	53.964	4	-1.1466
NODE_608_12	9	adenosylhomocysteinase	85.554	3	-0.41082
NODE_609_4	581	hypothetical protein	51.346	3	0.45698
NODE_609_8	814	putative het domain containing protein	86.833	3	-1.4288
NODE_611_13	80	clathrin heavy chain	69.331	4	3.43
NODE_611_14	644	related to dipeptidyl aminopeptidase B	56.258	3	-0.12584
NODE_616_12	115	gpi-anchored cell surface glycoprotein	41.876	3	-0.1656
NODE_616_3	404	NAD+ dependent glutamate dehydrogenase	53.567	2	-0.4171
NODE_620_7	182	hypothetical protein	104.89	2	-0.68335
NODE_621_11	223	hypothetical protein	46.892	3	4.164
NODE_623_13	21	hypothetical protein	61.344	3	4.1745
NODE_623_14	25	ribosomal protein L28e	45.829	3	-3.0946
NODE_623_14	93	ribosomal protein L28e	123.42	2	-0.30389
NODE_623_14	72	ribosomal protein L28e	82.452	2	-0.98683
NODE_623_14	108	ribosomal protein L28e	100.04	2	-1.0511
NODE_623_14	86	ribosomal protein L28e	70.089	3	-1.5716
NODE_623_14	100	ribosomal protein L28e	95.094	2	-0.66496
NODE_623_25	9	uncharacterized protein	61.444	3	1.288
NODE_623_25	15	uncharacterized protein	61.444	3	1.288
NODE_629_19	45	Flavin amine oxidase	53.013	2	1.8615
NODE_62_2	113	hypothetical protein	87.789	2	-2.0645
NODE_635_49	479	pyruvate carboxylase	44.318	2	-0.24029
NODE_635_49	1387	pyruvate carboxylase	48.216	3	4.2049
NODE_635_49	1458	pyruvate carboxylase	83.862	2	-0.020987
NODE_635_49	230	pyruvate carboxylase	62.466	3	-1.0582
NODE_635_49	1498	pyruvate carboxylase	79.148	3	0.85656
NODE_640_4	77	phospholipase PldA, putative	49.418	2	-0.14134
NODE_643_13	130	putative aldehyde dehydrogenase protein	42.317	3	-1.2621
NODE_643_13	190	putative aldehyde dehydrogenase protein	78.244	3	-1.4475
NODE_646_11	419	methylcitrate synthase precursor	97.431	2	-0.33921
NODE_646_15	125	hypothetical protein	72.2	2	-1.207
NODE_648_3	88	putative adenylate kinase (ATP-AMP transphosphorylase)	48.442	2	-1.138
NODE_648_3	208	putative adenylate kinase (ATP-AMP transphosphorylase)	110.38	2	-1.1004
NODE_648_3	211	putative adenylate kinase (ATP-AMP transphosphorylase)	59.728	3	-0.29485
NODE_650_17	137	ATP synthase subunit 4	66.267	2	-2.5344
NODE_650_20	44	uncharacterized protein	44.511	2	-0.84099
NODE_650_4	26	hypothetical protein	64.485	3	-0.21805
NODE_652_4	59	hypothetical protein	80.871	2	-0.5792
NODE_653_10	402	stomatin-like protein	42.976	2	-0.79674
NODE_653_6	55	Ribosomal protein S2, eukaryotic/archaeal	71.98	3	-0.28152
NODE_65_7	156	hypothetical protein	48.701	3	-0.59885
NODE_665_3	41	predicted protein	43.761	3	-1.6621
NODE_66_6	269	predicted protein	50.484	3	0.51066
NODE_672_20	156	zinc knuckle domain containing protein	73.386	3	0.090765
NODE_672_20	72	zinc knuckle domain containing protein	47.058	4	-1.3485
NODE_67_37	72	catalase/peroxidase HPI	67.08	2	0.56331

NODE_67_37	590	catalase/peroxidase HPI	40.616	3	1.3434
NODE_67_37	122	catalase/peroxidase HPI	41.448	3	0.51082
NODE_67_37	746	catalase/peroxidase HPI	40.767	2	-0.052078
NODE_67_40	117	hypothetical protein	68.224	3	-0.28156
NODE_681_12	742	L-aminoadipate-semialdehyde dehydrogenase	47.603	2	-1.5688
NODE_682_12	46	uncharacterized protein	77.062	2	-2.7375
NODE_682_12	37	uncharacterized protein	95.094	2	-1.5569
NODE_689_14	90	glucose-6-phosphate 1-dehydrogenase	40.496	2	-2.1856
NODE_689_14	229	glucose-6-phosphate 1-dehydrogenase	43.861	3	3.9575
NODE_689_14	307	glucose-6-phosphate 1-dehydrogenase	76.073	2	-1.673
NODE_689_14	133	glucose-6-phosphate 1-dehydrogenase	60.255	4	-1.0485
NODE_689_29	385	hypothetical protein	49.425	3	2.7898
NODE_689_32	312	hypothetical protein	79.116	3	0.029555
NODE_689_37	398	60S ribosomal protein L2	41.621	4	4.4135
NODE_689_38	142	putative g2 mitotic-specific cyclin-b protein	87.149	3	-0.41617
NODE_690_16	60	hypothetical protein	107.79	2	-0.59004
NODE_690_2	213	6-phosphogluconolactonase	66.267	2	1.5505
NODE_690_2	218	6-phosphogluconolactonase	64.121	3	-0.30399
NODE_696_4	174	molybdenum cofactor synthesis domain-containing protein	74.301	3	-1.2127
NODE_699_19	384	dicarboxylic amino acid permease	41.088	3	-1.0089
NODE_699_28	31	predicted protein	75.611	4	2.86
NODE_6_10	391	phenylalanyl-tRNA synthetase,beta subunit	61.03	3	-0.90409
NODE_6_10	156	phenylalanyl-tRNA synthetase, beta subunit	43.794	3	-0.24507
NODE_6_11	389	hypothetical protein	91.867	3	1.8478
NODE_6_16	385	hypothetical protein	51.286	2	0.18639
NODE_6_19	1256	MIF4-like, type 1/2/3	45.28	4	-0.76455
NODE_6_8	10	40S ribosomal protein S3	85.355	3	-0.60262
NODE_6_8	111	40S ribosomal protein S3	70.552	3	-1.3359
NODE_6_8	78	40S ribosomal protein S3	88.37	3	-1.0781
NODE_714_1	412	related to vacuolar ATP synthase subunit H	44.863	3	0.03897
NODE_71_11	187	predicted protein	118.28	2	-0.44234
NODE_71_6	125	peroxisomal dehydratase	62.617	4	-0.25003
NODE_729_3	50	40S ribosomal protein S23	58.98	4	0.55914
NODE_729_3	39	40S ribosomal protein S23	75.018	3	-0.090529
NODE_729_3	78	40S ribosomal protein S23	58.981	2	-0.58958
NODE_72_21	60	Ribosomal protein L22/L17,eukaryotic/archaeal	91.961	3	0.70324
NODE_72_21	132	Ribosomal protein L22/L17,eukaryotic/archaeal	66.664	3	0.62219
NODE_730_1	35	guanine nucleotide-binding protein	40.187	3	-1.7706
NODE_730_1	141	guanine nucleotide-binding protein	51.066	2	0.23321
NODE_730_1	51	guanine nucleotide-binding protein	66.27	2	2.2381
NODE_730_4	50	predicted protein	67.646	4	-1.1352
NODE_730_4	64	predicted protein	78.69	5	-3.4861
NODE_730_4	458	predicted protein	43.592	3	0.38694
NODE_730_4	341	predicted protein	78.324	3	-0.80439
NODE_732_3	447	methylcrotonoyl-CoA carboxylase beta chain	53.756	3	0.7321
NODE_732_3	177	methylcrotonoyl-CoA carboxylase beta chain	44.543	2	-1.8554
NODE_733_10	172	fatty acid synthase subunit alpha	73.885	3	-0.44135
NODE_733_10	64	fatty acid synthase subunit alpha	90.614	2	0.0151
NODE_733_10	58	fatty acid synthase subunit alpha	45.614	3	-2.4803
NODE_733_11	542	fatty acid synthase subunit alpha	109.66	3	-0.41717
NODE_733_11	1288	fatty acid synthase subunit alpha	52.599	3	-0.083227
NODE_733_11	880	fatty acid synthase subunit alpha	66.692	2	-1.1393
NODE_733_11	680	fatty acid synthase subunit alpha	66.023	3	-1.7782
NODE_733_11	282	fatty acid synthase subunit alpha	61.444	3	-0.82023
NODE_733_11	853	fatty acid synthase subunit alpha	40.941	3	2.7391
NODE_733_11	1560	fatty acid synthase subunit alpha	63.29	2	0.35143

NODE_733_11	1396	fatty acid synthase subunit alpha	52.185	3	-0.83404
NODE_733_17	406	ferrochelatase precursor	79.659	2	0.24544
NODE_733_19	509	cell division control protein 3	74.464	3	-0.73811
NODE_733_9	920	fatty acid synthase beta subunit dehydratase	71.451	2	-0.64399
NODE_733_9	889	fatty acid synthase beta subunit dehydratase	71.614	3	-1.6264
NODE_733_9	1450	fatty acid synthase beta subunit dehydratase	73.833	3	-1.1838
NODE_73_16	60	predicted protein	67.952	2	0.79801
NODE_73_26	236	hypothetical protein	78.814	2	-0.36491
NODE_73_36	104	40S ribosomal protein S12	61.815	3	-1.1291
NODE_73_36	66	40S ribosomal protein S12	84.738	3	-0.076121
NODE_73_36	138	40S ribosomal protein S12	49.592	3	-1.6852
NODE_747_21	267	26S proteasome regulatory subunit RPN5	74.92	3	-1.1216
NODE_747_21	257	26S proteasome regulatory subunit RPN5	45.614	4	0.71211
NODE_74_15	205	Hsp70 family protein	52.247	3	-0.38733
NODE_74_15	190	Hsp70 family protein	46.88	3	-0.72957
NODE_74_15	21	Hsp70 family protein	62.546	2	-0.71653
NODE_74_22	49	hypothetical protein	84.169	2	0.020387
NODE_74_22	36	hypothetical protein	67.385	4	0.3439
NODE_74_22	70	hypothetical protein	56.719	3	-1.0863
NODE_74_22	29	hypothetical protein	66.692	2	-0.44513
NODE_74_22	41	hypothetical protein	92.151	3	-1.2653
NODE_74_23	254	hypothetical protein	58.699	2	-0.16223
NODE_74_23	259	hypothetical protein	61.161	3	0.5737
NODE_74_23	290	hypothetical protein	67.08	5	4.4004
NODE_74_7	372	delta 1-pyrroline-5-carboxylate dehydrogenase	53.751	2	0.11645
NODE_74_7	555	delta 1-pyrroline-5-carboxylate dehydrogenase	43.68	3	1.104
NODE_75_49	33	hypothetical protein	44.98	3	-0.49278
NODE_76_46	206	GTP cyclohydrolase 1	44.252	3	4.0471
NODE_76_52	62	Glucosamine 6-phosphate N-acetyltransferase	56.404	2	-0.1181
NODE_774_5	397	hypothetical protein	72.2	3	-0.20044
NODE_779_1	4	Glyceraldehyde-3-phosphate dehydrogenase	55.839	3	-0.068314
NODE_779_1	253	Glyceraldehyde-3-phosphate dehydrogenase	108.56	3	1.5748
NODE_779_1	213	Glyceraldehyde-3-phosphate dehydrogenase	64.121	3	0.55797
NODE_779_1	101	Glyceraldehyde-3-phosphate dehydrogenase	63.302	3	-0.47877
NODE_779_1	111	Glyceraldehyde-3-phosphate dehydrogenase	122.52	3	-1.1688
NODE_779_1	188	Glyceraldehyde-3-phosphate dehydrogenase	44.614	3	0.42429
NODE_77_9	154	serine/threonine-protein phosphatase pp2a catalytic subunit	102.07	2	2.795
NODE_795_1	557	putative tryptophan -dioxygenase protein	52.576	2	0.2379
NODE_795_1	719	putative tryptophan -dioxygenase protein	54.09	2	-0.95266
NODE_79_12	24	Ribosomal protein L44e	53.775	3	-0.38435
NODE_79_12	19	Ribosomal protein L44e	79.659	3	0.028511
NODE_79_18	82	Transcriptional Coactivator p15 family protein	65.252	3	0.53009
NODE_79_23	636	leukotriene A-4 hydrolase	80.69	2	1.3145
NODE_79_9	100	Multiprotein-bridging factor 1	48.981	2	0.10759
NODE_7_18	260	putative alcohol dehydrogenase protein	51.726	2	-0.56622
NODE_801_1	356	hypothetical protein	40.137	5	-1.5255
NODE_801_1	243	hypothetical protein	90.05	2	-0.40987
NODE_801_1	84	hypothetical protein	66.989	3	-0.53836
NODE_804_31	596	Oligopeptide transporter	71.263	3	-0.25027
NODE_804_31	318	Oligopeptide transporter	93.766	3	-1.6643
NODE_804_31	307	Oligopeptide transporter	63.29	3	-1.469
NODE_828_7	481	aldehyde dehydrogenase	65.627	2	0.42665
NODE_82_12	124	calreticulin	50.354	3	0.43441
NODE_82_12	197	calreticulin	67.334	3	-1.6503
NODE_82_12	434	calreticulin	47.774	3	-0.26574
NODE_82_12	190	calreticulin	56.205	2	0.014734

NODE_82_13	183	putative succinate dehydrogenase flavo protein	100.93	2	0.42793
NODE_82_20	360	Inorganic pyrophosphatase	66.267	2	-2.0728
NODE_82_20	217	Inorganic pyrophosphatase	53.448	3	-0.51299
NODE_82_20	381	Inorganic pyrophosphatase	63.235	3	-1.365
NODE_82_20	341	Inorganic pyrophosphatase	79.489	4	1.9794
NODE_82_23	319	hexokinase	47.712	3	1.5623
NODE_82_23	337	hexokinase	54.225	4	2.403
NODE_82_23	26	hexokinase	76.073	3	-0.14112
NODE_82_25	240	vacuolar transporter chaperone 4	55.851	3	3.3253
NODE_82_52	635	UTP--glucose-1-phosphateuridylyltransferase	60.093	3	-1.3226
NODE_82_52	536	UTP--glucose-1-phosphateuridylyltransferase	55.353	4	0.36271
NODE_82_52	619	UTP--glucose-1-phosphateuridylyltransferase	45.28	3	-0.20065
NODE_82_52	351	UTP--glucose-1-phosphateuridylyltransferase	71.148	3	-1.9198
NODE_82_52	422	UTP--glucose-1-phosphate uridylyltransferase	60.255	3	0.22693
NODE_83_19	323	predicted protein	77.732	3	3.1275
NODE_83_2	224	hexokinase	82.261	2	-0.74926
NODE_83_21	1066	CAMK/CAMKL/GIN4 protein kinase	53.493	2	-0.25097
NODE_83_34	93	hypothetical protein	66.92	2	1.0221
NODE_83_4	431	hypothetical protein FOXB_11752	51.445	2	-0.37207
NODE_83_45	184	hypothetical protein	56.404	3	1.4635
NODE_83_48	283	aspartate-semialdehyde dehydrogenase	65.423	3	-0.64459
NODE_841_1	467	hypothetical protein	51.727	2	3.0052
NODE_848_14	75	ThiJ/PfpI family protein	100.88	2	-0.22453
NODE_848_14	73	ThiJ/PfpI family protein	74.611	3	-2.5783
NODE_84_8	479	putative aldehyde dehydrogenase protein	45.28	2	-2.0917
NODE_84_8	246	putative aldehyde dehydrogenase protein	64.103	3	-1.6003
NODE_84_8	304	putative aldehyde dehydrogenase protein	96.229	3	0.43213
NODE_84_8	189	putative aldehyde dehydrogenase protein	88.427	3	-1.2286
NODE_84_8	235	putative aldehyde dehydrogenase protein	86.288	3	0.27403
NODE_859_2	65	vacuolar sorting receptor (Mrl1), putative	53.756	3	0.48239
NODE_85_10	39	predicted protein	74.841	2	-0.74725
NODE_85_10	40	predicted protein	71.176	2	0.33612
NODE_85_10	255	predicted protein	73.499	2	-0.1308
NODE_85_16	107	DNA replication licensing factor mcm5	62.633	2	0.24516
NODE_85_3	419	hypothetical protein	54.402	3	0.29447
NODE_872_1	323	hypothetical protein	55.196	2	-0.08707
NODE_872_4	103	c30d10.14-like protein	66.267	2	-0.43253
NODE_872_4	108	c30d10.14-like protein	46.249	3	0.051552
NODE_875_3	312	sodium/phosphate symporter, putative	66.636	3	2.9676
NODE_875_3	338	sodium/phosphate symporter,putative	56.432	2	-0.24281
NODE_875_3	400	sodium/phosphate symporter, putative	85.676	3	-1.929
NODE_875_5	8	hypothetical protein	136.5	2	0.17481
NODE_883_1	116	F-type H+-transporting ATPase subunit epsilon	66.267	3	-0.38108
NODE_89_14	131	predicted protein	86.497	3	0.67604
NODE_89_19	587	formate dehydrogenase	41.908	5	2.801
NODE_89_37	40	predicted protein	82.287	2	-0.7523
NODE_89_44	262	trimethyllysine dioxygenase family protein	51.762	2	0.34976
NODE_89_51	71	predicted protein	42.083	3	-0.88229
NODE_8_18	137	uncharacterized protein	53.034	3	-0.31938
NODE_8_6	60	putative phospholipase C	90.614	3	-0.60859
NODE_902_3	148	hypothetical protein	42.958	3	0.48336
NODE_905_2	353	uncharacterized protein	68.49	3	-1.2975
NODE_905_2	110	uncharacterized protein	75.548	3	-0.12609
NODE_905_2	82	uncharacterized protein	51.31	3	-1.9394
NODE_912_5	939	histidine kinase	79.659	2	-0.34034
NODE_918_2	52	Amino-acid permease inda1	77.062	3	-0.95878

NODE_918_2	68	Amino-acid permease inda1	70.26	4	0.32362
NODE_920_4	65	hypothetical protein	43.68	2	-0.70189
NODE_920_7	407	XPG I-region protein	77.058	3	-0.3085
NODE_920_8	610	cell wall biogenesis protein phosphatase Ssd1	86.472	3	-0.39484
NODE_921_11	151	hypothetical protein	64.207	3	0.9577
NODE_921_11	264	hypothetical protein	81.625	3	-1.3935
NODE_924_25	33	predicted protein	52.482	3	-0.57186
NODE_924_34	134	Tyrosine-protein phosphatase YVH1	40.493	3	-0.18139
NODE_926_2	170	flavin-binding monooxygenase-like protein	76.679	3	0.37618
NODE_926_2	347	flavin-binding monooxygenase-like protein	47.037	3	0.0047377
NODE_944_25	246	hypothetical protein	68.132	2	-1.2705
NODE_944_25	251	hypothetical protein	83.182	2	1.0544
NODE_944_25	328	hypothetical protein	79.489	3	-0.20693
NODE_944_25	71	hypothetical protein	62.861	3	-0.21801
NODE_944_25	424	hypothetical protein	47.603	3	-1.2201
NODE_944_25	88	hypothetical protein	42.947	3	-1.4806
NODE_944_25	93	hypothetical protein	65.224	3	-1.5673
NODE_944_25	77	hypothetical protein	51.726	2	0.3558
NODE_944_25	56	hypothetical protein	62.847	3	-1.5607
NODE_944_25	325	hypothetical protein	73.499	2	1.1833
NODE_944_26	564	predicted protein	69.721	3	-0.73965
NODE_95_16	330	hypothetical protein	51.066	2	-4.4182
NODE_95_26	26	Hsp90 co-chaperone AHA1	79.089	3	-0.33216
NODE_967_2	283	predicted protein	60.062	4	3.5134
NODE_967_2	329	predicted protein	72.582	3	-0.89952
NODE_968_3	89	hypothetical protein	69.815	3	-1.49
NODE_968_3	31	hypothetical protein	53.565	4	3.2202
NODE_969_16	125	hypothetical protein	42.21	3	-0.50657
NODE_96_10	338	hypothetical protein	58.98	3	-0.16719
NODE_96_4	132	T-complex protein 1 subunit gamma	63.565	3	-1.3637
NODE_973_5	230	hypothetical protein	69.598	3	0.41296
NODE_97_11	422	related to BUD7 protein	56.432	3	-1.7265
NODE_97_6	18	hypothetical protein	44.425	2	-3.2135
NODE_985_3	43	hypothetical protein	64.104	4	-0.89118
NODE_987_10	286	glycoside hydrolase family 72 protein	58.172	2	-0.79409
NODE_987_11	87	predicted protein	86.189	3	-1.2008
NODE_987_11	77	predicted protein	76.228	2	-0.9783
NODE_987_11	83	predicted protein	54.259	3	-1.5719
NODE_987_11	442	predicted protein	46.88	3	-1.0192
NODE_987_11	193	predicted protein	60.489	3	-0.27825
NODE_987_12	35	ATP-citrate synthase subunit 1	46.892	3	-1.8662
NODE_987_12	536	ATP-citrate synthase subunit 1	61.344	4	-0.72151
NODE_987_12	342	ATP-citrate synthase subunit 1	61.56	2	3.1301
NODE_987_12	153	ATP-citrate synthase subunit 1	50.04	3	1.593
NODE_987_12	616	ATP-citrate synthase subunit 1	58.32	3	-1.5276
NODE_987_8	137	hypothetical protein	61.161	2	1.1707
NODE_987_8	120	hypothetical protein	95.362	2	-2.2198
NODE_987_8	228	hypothetical protein	57.288	3	0.11481
NODE_987_8	117	hypothetical protein	51.445	2	-0.32746
NODE_98_11	119	ThiJ/PfpI family protein	40.187	3	4.0542
NODE_98_38	437	elongation factor 2	40.941	3	-0.38153
NODE_98_38	632	elongation factor 2	56.514	3	-0.54353
NODE_98_38	558	elongation factor 2	61.11	2	-0.66778
NODE_98_38	322	elongation factor 2	62.338	4	1.2882
NODE_98_38	491	elongation factor 2	77.662	3	0.81883
NODE_98_38	644	elongation factor 2	55.676	3	-2.1238

NODE_98_38	379	elongation factor 2	40.496	3	-0.36582
NODE_98_38	318	elongation factor 2	73.927	4	-0.7768
NODE_98_38	329	elongation factor 2	102.28	2	0.49915
NODE_98_38	541	elongation factor 2	95.067	3	-0.10647
NODE_98_42	605	hypothetical protein	51.064	2	1.0225
NODE_98_44	42	cytochrome c oxidase subunit 6b	53.3	4	-0.51614
NODE_98_44	31	cytochrome c oxidase subunit 6b	40.968	4	-1.5695
NODE_98_46	38	uncharacterized protein	54.343	4	-0.20569
NODE_98_46	10	uncharacterized protein	65.224	3	-0.32998
NODE_98_46	31	uncharacterized protein	57.434	4	-0.70118
NODE_98_46	5	uncharacterized protein	52.867	3	-0.27653
NODE_98_52	92	NSF attachment protein	54.982	2	-1.8087
NODE_98_52	188	NSF attachment protein	58.63	2	-1.592
NODE_98_52	193	NSF attachment protein	41.621	4	0.03418
NODE_98_52	184	NSF attachment protein	54.859	3	4.3782
NODE_98_69	118	hypothetical protein	79.652	3	-1.6257
NODE_98_69	139	hypothetical protein	95.428	2	-0.81456
NODE_98_69	336	hypothetical protein	93.374	3	-0.65435
NODE_98_70	608	hypothetical protein	94.122	3	0.20467
NODE_98_70	338	hypothetical protein	44.511	3	0.7182
NODE_98_81	556	putative acetyl-coenzyme a synthetase protein	66.351	3	0.65114
NODE_990_7	266	predicted protein	42.314	3	4.2929
NODE_990_7	268	predicted protein	94.717	3	-0.29002
NODE_997_7	82	hypothetical protein FOXB_06063	86.898	2	0.6572
NODE_998_3	120	dihydroxy-acid dehydratase	51.346	3	-0.11757
