# Supplementary material for: Tandem Mass Tag labelling quantitative acetylome analysis of differentially modified proteins during mycoparasitism of Clonostachys chloroleuca 67–1
Source: Sci Rep. 2021 Nov 17;11:22383. doi: 10.1038/s41598-021-01956-2 (PMC8599485; doi:10.1038/s41598-021-01956-2)
Supplement: Supplementary file 3 — Supplementary Information 3. [file 41598_2021_1956_MOESM3_ESM.pdf]

Table S2. Motifs Identified in the *C. rosea* 67-1 Acetylome

motif logo	motif	score	foreground		background		fold increase
			match	size	match	size	
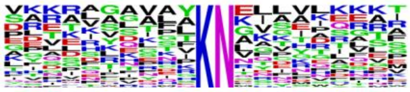	xxxxxxxxxx_K_Nxxxxxxx<br>xx	16.00	127	1448	13952	375368	2.4
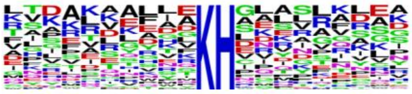	xxxxxxxxxx_K_Hxxxxxxx<br>xx	16.00	105	1321	9148	361416	3.1
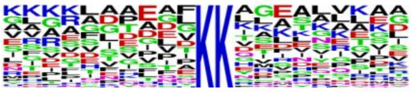	xxxxxxxxxx_K_Kxxxxxxx<br>xx	16.00	168	1216	24038	352268	2.0
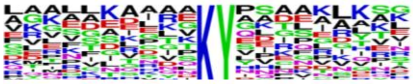	xxxxxxxxxx_K_Yxxxxxxx<br>xx	16.00	105	1048	10664	328230	3.1
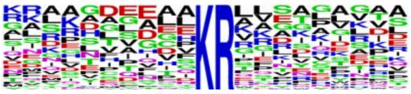	xxxxxxxxxx_K_Rxxxxxxxx<br>x	9.64	129	943	24442	317566	1.8
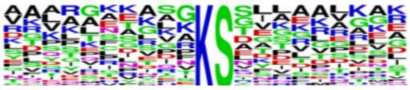	xxxxxxxxxx_K_Sxxxxxxxx<br>x	10.79	140	814	28218	293124	1.8
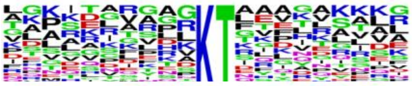	xxxxxxxxxx_K_Txxxxxxxx<br>x	8.83	105	674	22491	264906	1.8
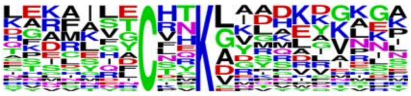	xxxxxxxCxx_K_xxxxxxxxx<br>x	8.19	26	569	2811	242415	3.9
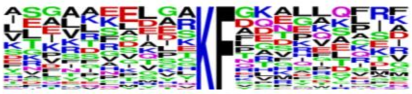	xxxxxxxxxx_K_Fxxxxxxxx<br>x	8.05	59	543	11560	239604	2.3
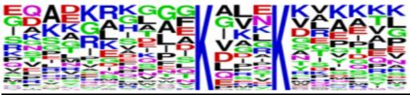	xxxxxxxxxx_K_xxxKxxxx<br>xx	8.32	66	484	14415	228044	2.2
